# Hyperosmolarity adversely impacts recombinant protein synthesis by *Yarrowia lipolytica*—molecular background revealed by quantitative proteomics

**DOI:** 10.1007/s00253-021-11731-y

**Published:** 2021-12-16

**Authors:** Monika Kubiak-Szymendera, Bozena Skupien-Rabian, Urszula Jankowska, Ewelina Celińska

**Affiliations:** 1 Department of Biotechnology and Food Microbiology, Poznan University of Life Sciences, Wojska Polskiego 48, 60-627 Poznan, Poland; 2 Proteomics and Mass Spectrometry Core Facility, Malopolska Centre of Biotechnology, Jagiellonian University, Gronostajowa 7a, 30-387 Krakow, Poland

**Keywords:** *Yarrowia lipolytica*, Heterologous protein, Proteomics of stress response

## Abstract

**Abstract:**

In this research, we were interested in answering a question whether subjecting a *Yarrowia lipolytica* strain overproducing a recombinant secretory protein (rs-Prot) to pre-optimized stress factors may enhance synthesis of the rs-Prot. Increased osmolarity (3 Osm kg^−1^) was the primary stress factor implemented alone or in combination with decreased temperature (20 °C), known to promote synthesis of rs-Prots. The treatments were executed in batch bioreactor cultures, and the cellular response was studied in terms of culture progression, gene expression and global proteomics, to get insight into molecular bases underlying an awaken reaction. Primarily, we observed that hyperosmolarity executed by high sorbitol concentration does not enhance synthesis of the rs-Prot but increases its transcription. Expectedly, hyperosmolarity induced synthesis of polyols at the expense of citric acid synthesis and growth, which was severely limited. A number of stress-related proteins were upregulated, including heat-shock proteins (HSPs) and aldo–keto reductases, as observed at transcriptomics and proteomics levels. Concerted downregulation of central carbon metabolism, including glycolysis, tricarboxylic acid cycle and fatty acid synthesis, highlighted redirection of carbon fluxes. Elevated abundance of HSPs and osmolytes did not outbalance the severe limitation of protein synthesis, marked by orchestrated downregulation of translation (elongation factors, several aa-tRNA synthetases), amino acid biosynthesis and ribosome biogenesis in response to the hyperosmolarity. Altogether we settled that increased osmolarity is not beneficial for rs-Prots synthesis in *Y. lipolytica*, even though some elements of the response could assist this process. Insight into global changes in the yeast proteome under the treatments is provided.

**Key points:**

• Temp enhances, but Osm decreases rs-Prots synthesis by Y. lipolytica.

• Enhanced abundance of HSPs and osmolytes is overweighted by limited translation.

• Global proteome under Osm, Temp and Osm Temp treatments was studied.

**Supplementary Information:**

The online version contains supplementary material available at 10.1007/s00253-021-11731-y.

## Introduction

Environmental factors evoke multilayered responses in the yeast producer cells. Some treatment conditions are known to promote synthesis of specific compounds, while the others exert adverse impact in this regard. For example, it is widely recognized that hyperosmotic conditions promote synthesis of polyols in yeast and that decreased temperature is beneficial for synthesis and secretion of recombinant, secretory proteins (rs-Prots). Both these phenomena were also observed and employed in bioprocesses with a nonconventional yeast species *Yarrowia lipolytica* (Tomaszewska et al. 2012; Yang et al. 2014, 2015; Kubiak et al. 2021), which is a popular workhorse in numerous industrial applications (Groenewald et al. 2014; Madzak 2018, 2021). Exploitation of *Y. lipolytica* as a rs-Prot expression platform receives significant attention due to several advantageous qualities of this species predisposing it to high level production (Celińska and Nicaud 2019; Theron et al. 2020). Up to date, several strategies have been adopted to further enhance synthesis of rs-Prots in this species, including invention of potent cloning tools (Schwartz et al. 2016; Celińska et al. 2017b, 2018; Trassaert et al. 2017; Wong et al. 2017; Park et al. 2019), different bioprocessing and medium optimization approaches (Chang et al. 1998a,b; Gasmi et al. 2011a,b; Celińska et al. 2017a), to a recent co-expression of omics-targeted secretory helpers that assist synthesis and secretion of rs-Prots (Korpys-Woźniak et al. 2021; Korpys-Woźniak and Celińska 2021). In our current studies, we are interested in answering a question whether subjecting a *Y. lipolytica* production culture to a specific, pre-optimized stress factor may enhance synthesis of rs-Prots. While the fact of beneficial impact of decreased temperature on synthesis and secretion of rs-Prots by yeast is widely recognized and largely generalizable, the impact of elevated osmolarity on the production has not been systematically addressed. Previous evidence suggests that increased osmotic pressure applied to cultures of filamentous fungi triggered enhanced synthesis of native proteins (Fiedurek 1998). Likewise, exposure of bacteria to high salt, sorbitol (SORB) and betaine concentrations increased production of a protein to be crystallized (Oganesyan et al. 2007). The outcome was attributed to increased cellular levels of osmolytes and chaperones, exerting protective activity toward polypeptides. Similar suggestions stemmed from a work on a recombinant *Y. lipolytica* strain expressing a heterologous invertase gene (Lazar et al. 2011). While the relationship between osmotic stress and enhanced synthesis of polyols in *Y. lipolytica* is clearly understood and well-described at molecular level (Kobayashi et al. 2015; Rzechonek et al. 2018), the effect of the former on the production of rs-Prots requires further studies.

In our previous experiments, we tested a working hypothesis on the beneficial effect of elevated osmotic pressure on the production of rs-Prots in *Y. lipolytica* (Kubiak et al. 2019). The hyperosmotic conditions were implemented by the addition of several chemically different osmoactive compounds, including glycerol (GLY), SORB, sucrose and NaCl at different concentrations. The treatment was followed by short-term incubation and determination of specific productivity of a reporter glucoamylase. We observed that the effect was largely dependent on the osmolyte used. The most beneficial was the addition of GLY and SORB, when the osmolality reached > 2.5 Osm kg^−1^. But since the correlation between a factor “osmolality” and a factor “specific activity” was low (*r* = 0.48), it was clear that more in-depth studies are necessary. Earlier, Yang et al. (2015) studied the total proteome of wild type *Y. lipolytica* subjected to 70 g L^−1^ of NaCl (Osm = 4.15 Osm kg^−1^) to reveal molecular background of erythritol (ERY) synthesis under these conditions. Using 2D-PAGE (two-dimensional polyacrylamide gel electrophoresis) followed by MS (mass spectrometry) of selected spots, the authors identified 44 differentially abundant proteins (DAPs) and determined their abundance level based on comparative band intensity. The same methodology was used in studies on dimorphic transition (Morín et al. 2007) and specificities of different amino acids (AAs) metabolism (Mansour et al. 2009) in *Y. lipolytica*.

Previously, we used global quantitative proteomics (liquid chromatography with tandem mass spectrometry; LC–MS/MS) to reveal molecular identities involved in synthesis of rose-like odor—2-phenylethanol (Celińska et al. 2015b). In the present research, we took advantage of this sensitive and unbiased method to study the impact of hyperosmolarity on synthesis of rs-Prots by *Y. lipolytica*. Considering our previous findings on the advantageous effect of decreased temperature on rs-Prots synthesis by this yeast species (Kubiak et al. 2021), we included this variant alone or in combination with hyperosmolarity to study its effect at a proteome level. Altogether, this research provides new insight into the actual relationship between hyperosmolarity and rs-Prots synthesis in *Y. lipolytica* characterized by biochemical, transcriptional and proteomic studies.

## Materials and methods

### Recombinant strain

*Y. lipolytica* strain GGY237 (Korpys-Woźniak et al. 2020) used in this study was a Po1h-derivative (*MatA, ura3-302, xpr2-322, axp1-2*) overexpressing a heterologous alpha-amylase-encoding gene from *Sitophilus oryzae* (GenBank: KP027641.1; (Celińska et al. 2015a)) under the synthetic 4UASpTEF promoter. The strain was constructed via Golden Gate strategy, as described previously (Celińska et al. 2017a, b).

### Inoculum preparation

Precultures were developed from 15% GLY stocks stored at − 80 °C, plated on YPD agar medium containing [g L^−1^]: yeast extract (YE; Merck, USA), 10; bactopeptone (BP; Merck, Darmstadt, Germany), 20; glucose (POCH, Gliwice, Poland), 20; agar (BIOCORP, Warsaw, Poland), 20. Twenty-four-hour biomass was transferred into 1-L shake flasks containing 100 mL of YPG20 medium ([g L^−1^]: YE, 10; BP, 20; GLY, 20) and incubated at 30 °C with 250 rpm agitation in a rotary shaker (BIOSAN, ES-20, Riga, Latvia) for 23 h.

### Bioreactor cultures and treatment conditions

Batch cultures were performed in Minifors 2 bioreactors (Infors, HT, Bottmingen-Basel, Switzerland) with a total volume of 2.6 L and a working volume of 1 L, in YPG120 medium, composed as follows [g L^−1^]: YE, 10; BP, 20; GLY, 120. The bioreactors were equipped with two Rushton turbines and sensors for temperature, pH and dissolved oxygen. pH was adjusted to 5.5 and controlled by automatic addition of 30% NaOH and 10% H_2_SO_4_. The temperature was maintained at 28 °C throughout the culture, except for the periods of thermal treatment, where indicated. Stirring and aeration were set at 700 rpm and 2 vvm, respectively. Antifoam agent (Antifoam 204, Sigma-Aldrich, St. Louis, MO, USA) was added automatically, when needed.

**Fig. 1:**
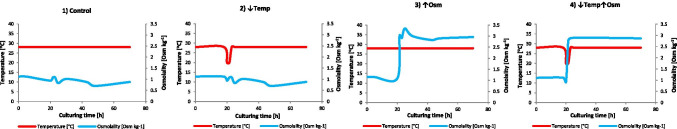
Treatment variants. Curves are based on actual measurements of temperature and osmolality read in real time by thermometer and in collected samples by osmometer, respectively. Y axis: temperature [°C]; auxiliary Y axis: osmolality [Osm kg^−1^], X axis: time [h]; 1) control variant, 2) ↓Temp variant, 3) ↑Osm variant, 4) ↓Temp↑Osm variant. Color code is explained in the legend. Given values are mean from biological quadruplicate ± SD

The treatments were executed starting from 20 h of culturing, which corresponds to the end of exponential/early stationary phase of growth. The cultures were conducted in four variants: (1) control (without treatment), (2; ↓Temp) with thermal treatment, (3; ↑Osm) with osmotic treatment, (4; ↓Temp↑Osm) with thermal and osmotic treatment (Fig. 1).

The applied thermal treatment (20 °C for 153.5 min) was previously optimized to maximize specific productivity of the target rs-Prot by *Y. lipolytica* (Kubiak et al. 2021). Hyperosmotic conditions (approx. 3.0–3.5 Osm kg^−1^) were also pre-optimized (not shown) and executed by aseptic addition of powdered SORB to reach the final concentration of 360 g L^−1^. Such manipulation does not transfer undesired microbiota to the culture, which was checked by surface plating (not shown). Noteworthy, at this concentration, SORB does not impact oxygen transfer rate, which was also verified. As demonstrated previously, SORB is not consumed by *Y. lipolytica* (Kubiak et al. 2019), and thus the implemented hyperosmolarity was stably maintained.

Each culture variant was conducted in four independent runs. Samples were collected throughout the culturing time, centrifuged (12,045 × *g*, 3 min; Eppendorf MiniSpin, Hamburg, Germany) and stored at − 20 °C (biomass and supernatant separately) to analyze the activity of the reporter protein, biomass accumulation, GLY consumption and synthesis of metabolites. Samples for total RNA and total protein isolation were collected at 23/25 h, centrifuged at 11,152 × *g* for 3 min at 4 °C (Eppendorf 5430 R, Eppendorf, Hamburg, Germany) and stored at − 80 °C until further analyzed. Each collected sample was analyzed in technical duplicate.

### Enzymatic assay

Activity of the extracellular alpha-amylase (SoAMY) was analyzed according to a microSIT protocol described previously (Borkowska et al. 2019). One activity unit (AU) corresponds to the amount of an enzyme that triggers decrease in the starch-iodine staining value equivalent to 1 mg of starch per 1 mL, during 1 min at pH 5.0 and 40 °C, under applied experimental conditions.

### Biomass concentration

Biomass concentration was determined using spectrophotometric measurements of absorbance at 600-nm wavelength (OD600). The biomass samples were first defrosted, washed twice in distilled water and suspended in water. All the samples were diluted 20–40 × , and 200 µL of them was transferred into a 96-well microplate. The absorbance was measured using a microplate reader (Multiskan Sky, Thermo Fisher Scientific Inc., Waltham, MA, USA). Subsequently, dry cellular weight (DCW) concentration was calculated according to a previously prepared standard equation (absorbance reading at 600 nm plotted against biomass concentration measured by a standard gravimetric method; gDCW L^−1^).

### Osmolality

Osmolality of culture medium was determined by a freezing‐point depression method using an osmometer (Marcel os3000, Marcel, Zielonka, Poland). The thawed supernatants were appropriately diluted (due to high GLY or SORB concentration) and subjected to the measurement. One osmolality unit was defined as the quantity of osmoles of the osmotically active substance per kilogram of the sample [Osm kg^−1^].

### Concentration of chemical compounds (high‐performance liquid chromatography, HPLC)

Concentration of small‐molecular weight metabolites including ERY, mannitol (MAN), citric acid (CA), alpha-ketoglutaric acid (aKG), externally added SORB and the carbon source (GLY) in the culturing medium was measured by HPLC. Defrosted and diluted supernatants were passed through 0.45-μm membrane syringe filters (Millipore, Merck-Millipore, Burlington, MA, USA). Analysis was conducted using a chromatograph VWR/Hitachi LaChrom Elite (Merck-Hitachi, Darmstadt, Germany), equipped with two detectors (RI L-2490 and UV L-2400) and a Rezex ROA 300 × 7.8 mm column (Phenomenex, Torrance, CA, USA), at 40 °C, under isocratic conditions, with a flow rate of 0.6 mL min^−1^ and 10 mM H_2_SO_4_, used as the mobile phase. Standard solutions of the chemicals were all purchased from Sigma-Aldrich (St. Louis, MO, USA). Quantitative analysis was performed in reference to the standard solutions (peak area) using EZChrom Elite (Agilent Technologies, Santa Clara, CA, USA) software.

### Expression level of stress-related genes using RTqPCR

Total RNA isolation, reverse transcription (RT) reaction and real-time quantitative PCR (RTqPCR) were conducted as described by Borkowska et al. (2020). Briefly, Bead-Beat Total RNA Mini kit (A&A Biotechnology, Gdynia, Poland) and MixerMill MM400 (Retsch GmbH, Haan, Germany) were used for isolation of RNA. TranScriba Kit (A&A Biotechnology, Gdynia, Poland) and a Veriti Thermal Cycler (Applied Biosystems, USA) were used for the first cDNA strand synthesis. The RTqPCR was carried out using SYBR®Green PCR MasterMix kit B (A&A Biotechnology, Poland), FrameStar® 96 Well Semi-Skirted PCR Plates and a 7500 Real-time PCR Thermalcycler (Applied Biosystems, Foster City, USA). Real-time PCR primers targeting the intracellular stress-related genes and the internal calibrator were designed with Primer Expert Software (Applied Biosystems, Foster City, USA) (Supplemental Table S1). Comparative gene expression analysis of the treated samples was conducted vs the control culture (Fig. 1). Data analysis was carried out according to the delta-delta Ct (ΔΔCt) method (Livak and Schmittgen 2001).

### Protein precipitation

The samples collected at 23/25 h of culturing were immediately centrifuged (as described above). The cellular pellets were resuspended in ice-cold breaking buffer (0.1 M sodium phosphate buffer, 5 μM DTT, 1 mM PMSF, 5% GLY) with glass beads (Sigma-Aldrich, St. Louis, MO, USA) and disrupted by repeated cycles (5 ×) of mixing at 30 strokes s^−1^ for 30 s in a MixerMill MM400 (Retsch GmbH, Haan, Germany) and incubation on ice for 1 min. The cellular debris were then separated by centrifugation (24,652 × *g*, 4 °C, 10 min; Eppendorf 5430 R; Eppendorf, Hamburg, Germany). The protein precipitation was carried out according to a standard methanol-chloroform method (Wessel and Flügge 1984). Briefly, the protein suspensions were combined with 4 volumes of methanol and 1 volume of chloroform, thoroughly mixed and centrifuged at 12,000 × *g* for 15 min. The aqueous phase was discarded, and organic phase was again mixed with 4 volumes of methanol, thoroughly mixed and centrifuged (12,000 × *g*, RT, 15 min). The supernatant was withdrawn, and the obtained protein pellets were air-dried. The protein pellet was resuspended in sterile 8 M urea/50 mM Tris–HCl/50 mM EDTA buffer (pH 8.0), according to Saito et al. (2019). The protein profile was examined for the protein integrity by sodium dodecyl sulphate polyacrylamide gel electrophoresis (SDS-PAGE) (Laemmli 1970). The protein samples were stored at − 80 °C until analyzed.

### Tryptic digestion and LC–MS/MS analysis

The samples were processed through a filter-aided sample preparation (FASP) method (Wiśniewski et al. 2009) as described previously (Pinski et al. 2021) with minor modifications. The protein samples (50 µg) were brought to 200 µL with urea solution (8 M urea, 50 mM ammonium bicarbonate). Proteins were digested overnight with trypsin (Promega, Madison, WI, USA) at an enzyme to protein ratio of 1:50. About 1 µg of the resulting peptide mixture was injected on a Q Exactive mass spectrometer (Thermo Fisher Scientific) coupled with a nanoHPLC (UltiMate 3000 RSLCnano System, Thermo Fisher Scientific, Waltham, MA, USA). The parameters of the LC–MS/MS analysis were the same as in Pinski et al. (2021). Briefly, peptides were resolved on a 50 cm analytical column (AcclaimPepMapRLSC C18, Thermo Fisher Scientific; ID 75 μm, particle size 2 μm, pore size 100 Å) in the presence of 0.05% formic acid (JT Baker, Phillipsburg, NJ, USA) using a linear gradient of acetonitrile (2–40%) for 240 min. The mass spectrometric measurement was performed in the data-dependent mode using the Top12 method. The MS and MS/MS spectra were acquired at resolutions of 70,000 and 17,500, respectively. The performance of the LC–MS/MS platform was monitored using the QCloud quality control system (Chiva et al. 2018).

### MS data processing and analysis

Raw proteomics data were processed using MaxQuant 1.6.7.0 with incorporated Andromeda search engine (Cox et al. 2011; Tyanova et al. 2016a) set to detect variable modifications (Oxidation—M, Acetyl—Protein N-term) and fixed modification (Carbamidomethyl—C). Identification was based on mapping to the UniProtKB *Y. lipolytica* reference proteome (accessed on 05.03.2021, 6454 entries). Default MaxQuant parameters were used. The false discovery rate (FDR) for the peptide and protein identification was set to 1%. Label free quantification (LFQ) was enabled. Statistical analysis was conducted using Perseus 1.6.15.0 (Tyanova et al. 2016b). Quality of data and overall profiles were analyzed by inspection of multi-scatter plots illustrating Pearson correlation, PCA analysis and hierarchical clustering of all the proteome profiles in quadruplicate. Differentially expressed proteins under the treatment conditions vs the control were defined by the results of Student’s *t* test, with correction based on permutation-based FDR (threshold FDR < 0.05 for each comparison). Protein filter was set at minimum 3/4 valid values in each group. Protein identification was restricted to at least 2 unique peptides. A protein was defined as DAP (differentially abundant protein) provided the fold change > 1.20 or <  − 1.20. The mass spectrometry data were deposited to the ProteomeXchange Consortium (Vizcaíno et al. 2014) via the MassIVE repository with the dataset identifier PXD029106.

### Bioinformatic tools and databases

Assignment of YALI identifiers, molecular functions and biological processes was done using GRYC (http://gryc.inra.fr/) and PANTHER databases (http://pantherdb.org/). Statistical overrepresentation test tool from PANTHER was used to indicate overrepresented classes of proteins within specified groups of up- and downregulated DAPs. Visualization of Venn diagrams was done using InteractiVenn (http://www.interactivenn.net/) online tool. The search for binding motifs within promoter regions of aa-tRNA synthetase-encoding genes was conducted using 500 bp regions upstream from the start codon and MEME online tool (https://meme-suite.org/meme/) with default settings (Bailey et al. 2015).

### Statistical analysis

All the results are expressed as mean values ± standard deviation (± SD) of four biological replicates, each analyzed in at least duplicate. Statistical significance of the differences between compared sets of data was analyzed using one‐way analysis of variance (Microsoft Excel 2013 software). The level of significance was set at *p* < 0.05.

## Results

### Batch culture progression under the treatment conditions

**Fig. 2:**
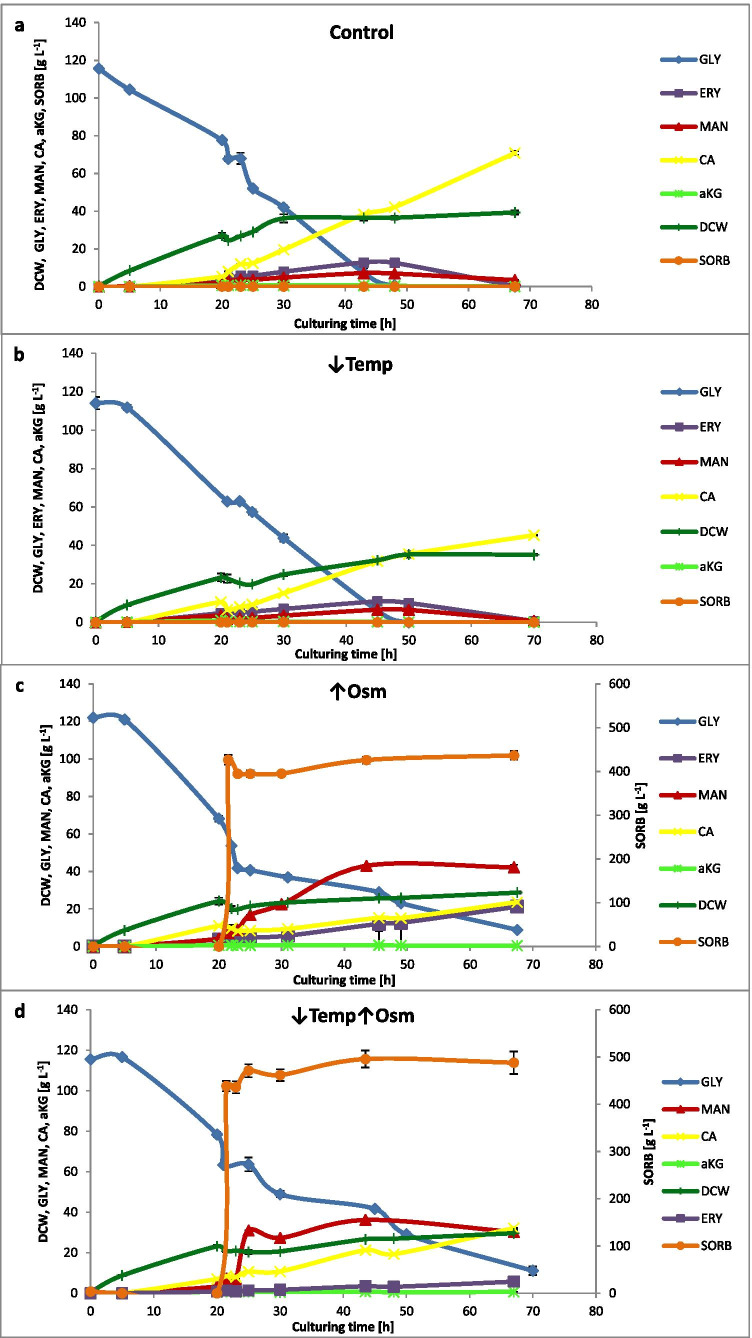
Growth (DCW), GLY utilization and metabolites synthesis over culturing time (Y axis and auxiliary Y axis: concentration in [g L^−1^]; X axis: time [h]) under different treatment variants and a control; **a**) control variant, **b**) ↓Temp variant, **c**) ↑Osm variant, **d**) ↓Temp↑Osm variant. Color code is explained in the legend. Abbreviations: GLY glycerol, ERY erythritol, MAN mannitol, CA citric acid, aKG alpha-ketoglutaric acid, DCW dry cellular weight, SORB sorbitol. Given values are mean from biological quadruplicate ± SD

Kinetics of batch cultivations in terms of GLY consumption, biomass formation and metabolites synthesis is given in Fig. 2. Until the treatment time, the cultures progression was highly corresponding in all the variants. Biomass productivity [dDCW dt^−1^] within this time range (0–20 h) was on average 1.35 g L^−1^ h^−1^. Following the treatment, the growth was largely slowed down. While in the control variant it continued up to 30 h reaching 1.25 g L^−1^ h^−1^, the exposure to ↓Temp decreased biomass productivity by more than half (equal to 0.45 g L^−1^ h^−1^ between 23 and 50 h). Increased osmolality of the culture medium led to further slowing down of growth (equal to 0.35 g L^−1^ h^−1^ between 23 and 50 h). The combined treatment ↑Osm↓Temp led to nearly complete arrest of biomass accumulation (equal to 0.07 g L^−1^ h^−1^ between 23 and 50 h). Until the treatment time, synthesis of metabolites was relatively low, and CA was the dominant molecule.

Following the ↓Temp treatment, GLY consumption, ERY and MAN synthesis remained unchanged vs the control. On the other hand, CA synthesis and growth were substantially reduced when compared to the control (~ 45 vs ~ 71 g L^−1^ of CA at the end of culture, in the ↓Temp and control, respectively). The perturbation with ↑Osm (alone or in combination) induced multiple physiological reactions. Expectedly, synthesis of polyols (especially MAN) was significantly enhanced reaching > 40 g L^−1^ in the ↑Osm variant. ERY was synthesized up to > 20 g L^−1^ in both ↑Osm and ↑Osm↓Temp. For both polyols, some minor fluctuations in the concentration were observed, which is typical for *Y. lipolytica* cultures. Notably, GLY was not fully consumed until the end of any ↑Osm-treated culture.

**Fig. 3:**
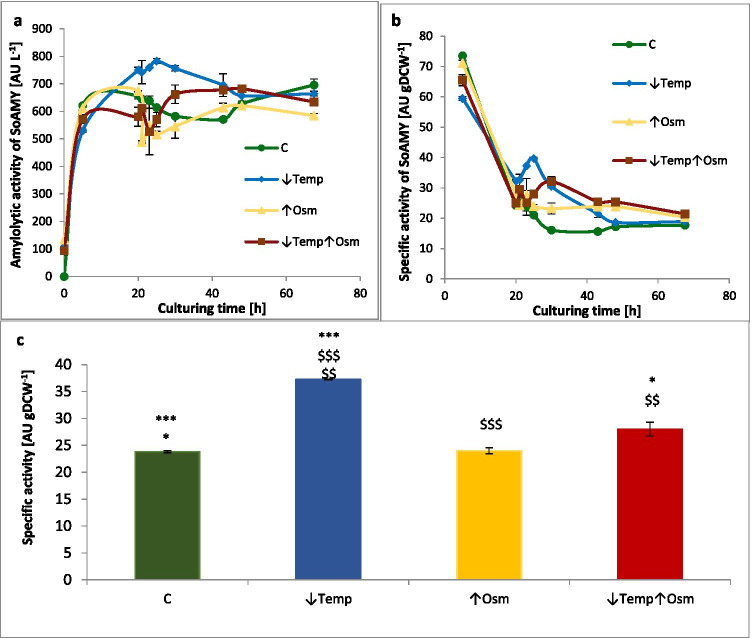
Amounts of extracellular activity of the rs-Prot (SoAMY) under different treatment variants and the control, given as: **a**) activity (titer) in [AU L^−1^] in time; **b**) specific activity normalized per biomass in [AU gDCW^−1^] in time; **c**) specific activity in [AU gDCW^−1^] at a time point of gene expression analysis and proteomics samples collection (23/25 h). Y axis: extracellular activity given in [AU L^−1^] or [AU gDCW^−1^]; X axis: **a**, **b**) time [h]; **c**) treatment variant. Color code is explained in the legend. Results indicate mean values from quadruplicate repetitions ± SD. In (**c**) *, $ indicate statistical significance with variant C (*), ↓Temp ($) at *p* value < 0.05, $$ at *p* value < 0.01, ***, $$$ at *p* value < 0.001

In terms of the reporter rs-Prot synthesis and secretion, the implemented perturbations had varying effects (Fig. 3). Temperature downshift had immediate (within 3 h) positive impact on the rs-Prot synthesis and secretion, improving its titer [AU L^−1^] by 1.2-fold and specific activity [AU gDCW^−1^] by 1.6-fold (*p* < 0.001). Macroscopically visible response to the ↑Osm was observable after > 5 h. After that time, the cells started to restore growth, synthesis of metabolites and the rs-Prot. In terms of specific activity at this time point, no significant difference could be seen between ↑Osm and the control. On the other hand, some minor but statistically significant improvement was observed for the ↑Osm↓Temp variant (by 18%; *p* < 0.05; Fig. 3c). As could be observed in Fig. 3b, both ↑Osm-treated variants turned out to be beneficial for AU gDCW^−1^ parameter upon prolonged exposure. However, this observation results rather from limited growth, especially in the case of the ↑Osm condition (compare Figs. 2 and 3a). In the following time range (30 to 48 h), the ↑Osm↓Temp variant reached plateau in terms of extracellular activity (Fig. 3a) at the averaged level of 674.5 AU L^−1^ (vs 593.8 AU L^−1^ for the control, 703.2 and 593.4 AU L^−1^ in ↓Temp and ↑Osm, respectively).

### Expression level of selected genes involved in temperature and hyperosmolarity stress response

**Fig. 4:**
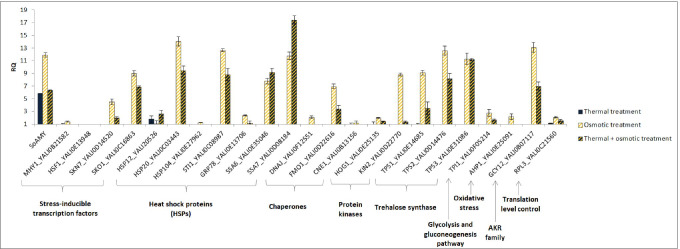
Expression level of selected genes analyzed by RTqPCR. Samples for gene expression analysis were collected at 23/25 h of culturing. Y axis: relative quantitation level determined by ΔΔCt analysis; X axis: analyzed genes—shortened name and YALI code. RQ relative quantitation. Shared/specific molecular functions of the genes are indicated. Color code is explained in the legend. Error bars indicate relative quantitation (RQ) ± SD from technical duplicates

Samples for molecular analyses were collected at the time point at which physiological response of the culture was observed (23 h for ↓Temp, and 25 h for ↑Osm and ↓Temp↑Osm). Gene expression analysis was conducted for a set of genes which, according to the literature, were shown responsive to either ↑Osm or ↓Temp in *Y. lipolytica* (Table 1). In addition, the heterologous gene encoding the reporter rs-Prot was also tracked for expression level. In contrast to what was seen macroscopically, the highest upregulation of the *SoAMY* gene was detected under ↑Osm (Fig. 4). Likewise, a number of the stress-related genes, including those involved in gene expression regulation (*Skn7* and *Sko1*), folding and maintenance in folding-competent state (*DnaJ*, *Fmo1*, HSPs), but also in synthesis of compatible solutes (*Tpss*, *Gcy12*) were significantly upregulated upon ↑Osm. ↓Temp treatment led to significant enhancement in *SoAMY* expression but negligible impact on expression of the stress response genes. Expression pattern of the genes was somehow similar under ↑Osm↓Temp as under ↑Osm, but in majority of cases the upregulation level was diminished due to the decreased temperature. Collectively, based on these data, it could be concluded that ↑Osm treatment imposed actual stress to the producer cells and awoke a transcriptional response. On the other hand, ↓Temp attenuated this response in the ↑Osm↓Temp variant. Sole ↓Temp did not impose any transcriptional reaction of the analyzed gene targets but enhanced expression of the heterologous gene under the synthetic promoter.

**Table 1:** List of genes analyzed by RTqPCR

Genes	YALI number	Function/effect of manipulation/response to stress conditions	Ref
*GCY12* aldo–keto reductase	YALI0B07117	NADPH-dependent conversion of carbonyl to hydroxyl groups; crucial role in responses to osmotic, oxidative and heat stress	(Yang et al. 2015)
*HSP12*	YALI0D20526	Low molecular weight heat shock proteins—role in folding, prevention of protein aggregation; especially high induction level under elevated temperature, as well as in conditions of osmotic and oxidative stress, or high concentration of alcohol, and at an early stationary phase of growth	(Yang et al. 2015)
*HSP20*	YALI0C03443		
*STI1*	YALI0C08987	70-kDa heat shock protein (HSP70) family; transcription induced by heat shock (28 → 37 °C), but not in response to osmotic and oxidative stress, and starvation; overrepresented in a proteome for the next 2 h at regeneration conditions (28 °C)	(Yang et al. 2015)
*GRP78* glucose regulated protein	YALI0E13706	70-kDa heat shock protein (HSP70) family; overexpression in conditions of heat shock (28 → 37 °C), oxidative stress; repression under osmotic stress; overrepresented in a proteome for the next 2 h at regeneration conditions (28 °C)	(Yang et al. 2015)
*HSP104*	YALI0E27962	Protein disaggregation, thermotolerance; dhsp104—high sensitivity to heat shock	(Verghese et al. 2012)
*DnaJ*	YALI0F12551	Mdj1p; mitochondrial co-chaperone; increased expression under hyperosmotic conditions	(Yang et al. 2015)
*Mhy1* (similar to Msn2/4 from *S. cerevisiae*)	YALI0B21582	The main transcription factor for stress response; it binds STRE sequence (CCCCT); significantly induced expression under heat shock (40 °C)	(Flores et al. 2011)
*HSF1* heat-shock factor 1	YALI0E13948	Constitutive activation of the HSR (heat-shock response)	(Hou et al. 2013)
*TPS1* trehalose-6-phosphate synthase	YALI0E14685	Genes involved in oligo- and polysaccharides metabolism; increased expression of TPS2 and TPS3 under heat shock; important role in cellular response to stress	(Flores et al. 2011)
*TPS2* trehalose-6-phosphatase	YALI0D14476		
*TPS3* subunit of trehalose synthase complex	YALI0E31086		
*CNE1*	YALI0B13156	Chaperone; high expression level induced by overexpression of HSF1 in *S. cerevisiae*; role in folding of glycoproteins, formation of glycosydic bonds (calnexin 1)	(Hou et al. 2013)
*FMO1*	YALI0D22616	Chaperone; high expression level induced by overexpression of HSF1 in *S. cerevisiae*; flavin-containing monooxygenase localized to the cytoplasmic side of the ER membrane, catalyses oxidation of thiol groups	(Hou et al. 2013)
*SSA6*	YALI0E35046	Cytosolic HSP70; increased expression under heat shock (42 °C for 1 h, and 37 °C for several hours)	(Sharma et al. 2009)
*SSA7*	YALI0D08184		
*HOG1*	YALI0E25135	Ser/Thr protein kinase belonging to MAPK family, involved in HOG (high-osmolarity glycerol) pathway, inducing response to osmotic stress, heat shock, acid stress and cold shock	(Thevenieau et al. 2007);
(Aguilera et al. 2007)			
*TPI1* triose-phosphate isomerase	YALI0F05214	Glycolytic enzyme catalyzing conversion of DHAP to GAP-3-P; approx. threefold increase in expression level under osmotic shock induced by NaCl	(Yang et al. 2015)
*AHP1* peroxiredoxin (PRX5*-*like subfamily)	YALI0E25091	Crucial role in oxidative stress—inactivation of reactive oxygen species; more than twofold increase in expression level under osmotic shock induced by NaCl	
*SKN7*	YALI0D14520*	Transcription factor involved in protein secretion, activation of response to oxidative and osmotic stress in *S. cerevisiae*; induction of HSPs in environmental stress response (*50% of similarity to NP_012076.3)	(Hohmann 2002)
*SKO1*	YALI0C16863**	Transcription factor involved in HOG pathway in *S. cerevisiae* (**53% of similarity to NP_014232*.*1)	
*KIN2*	YALI0D22770	Ser/Thr protein kinase; previously used as an efficient secretion helper, increasing the target protein production by 2.2-fold upon co-expression	(Gasser et al. 2007)
*RPL3*	YALI0C21560	Protein component of the large (60S) ribosomal subunit—as a control of the level of translation in cells	NCBI

### Treatment-induced proteomic changes—phenomena shared between the treatment variants

**Fig. 5:**
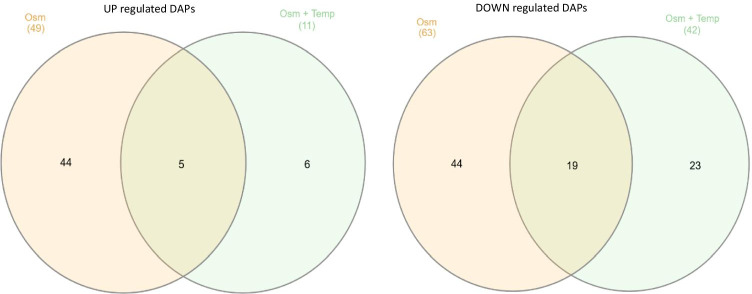
Venn diagrams representing shared and distinct DAPs that were up (left diagram) or down (right diagram) regulated in response to ↑Osm and ↑Osm↓Temp. Number of DAPs within a specific group is indicated. Allocation of specific DAPs to each group is given in Online Resource Table S4

Gene expression profiles did not explain macroscopic observations. Therefore, in the next step, we conducted high-throughput quantitative proteomics analysis to get more insight into molecular bases of the observed physiological responses. The analysis was conducted on biological quadruplicate samples. The replicates showed high level of Pearson correlation, and they were grouped together in PCA analysis and in hierarchical clustering (Supplemental Table S2). On average, 2330 ± 93 proteins were identified in each sample (median: 2339). DAPs were indicated based on Student’s *t* test and FDR correction for multiple comparisons. DAPs were then filtered for FC > 1.20 or <  − 1.20. The applied data processing pipeline yielded no DAPs upon comparison of ↓Temp vs control. Implementation of ↑Osm alone yielded 63 downregulated and 49 upregulated DAPs, while in combination (↑Osm↓Temp) resulted in 42 downregulated and 11 upregulated DAPs. Detailed lists of all the DAPs with functional annotation and fold change (FC) of abundance are given in Supplemental Table S3. Identification of common and unique DAPs responsive to ↑Osm and ↑Osm↓Temp was conducted by Venn diagram analysis. The results are shown in Fig. 5, and a detailed list of the members of each specific group is given in Supplemental Table S4. Among the uniformly upregulated DAPs, we identified three aldo–keto reductases (AKRs), including erythrose reductases Gcy12 (B07117) and Gcy13 (A15906). The commonly downregulated DAPs covered i.a. proteins involved in glycolysis (Pyc1/C24101), tricarboxylic acid cycle (TCA) (Acl1/E34793, Acl2/D24431), fatty acid (FA) metabolism (Fas1/B15059, Fas2/B19382, Pox3/D24750, Aal7/E20405), and proteins involved in proteolysis (F20592, C10494), AAs metabolism (B21846, A21417, D25168, C22088) and translation (a group of aa-tRNA synthetases, isoleucyl-/A00264, prolyl-/E05027, asparaginyl-/E05005).

**Fig. 6:**
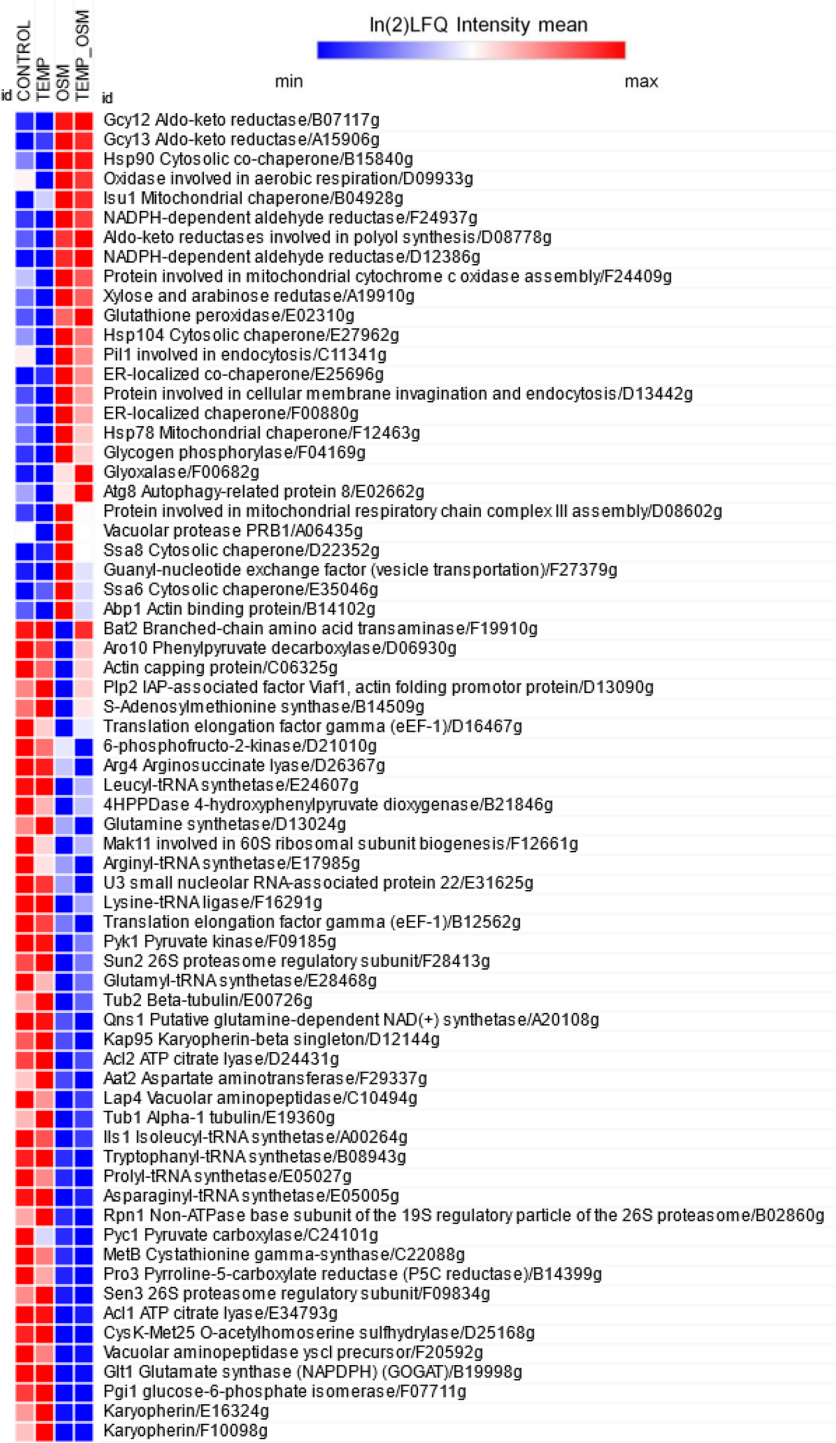
Heat map of selected DAPs. Up or down-representation of a given protein is color-coded according to the provided legend based on log2(LFQ Intensity) mean value from quadruplicate. Columns represent specific treatment conditions indicated at the top of the heat map. Rows represent individual DAPs indicated on the right site of the heat map. The heat map was prepared using Morpheus online tool (https://software.broadinstitute.org/morpheus/). Specific DAPs are given with their shortened name, function and shortened YALI code

The set of upregulated DAPs shared between ↑Osm and ↑Osm↓Temp (5 DAPs) was significantly enriched in molecular function alditol:NADP + 1-oxidoreductase activity, represented by 3 DAPs (per 13 total in the *Y. lipolytica* reference proteome; enrichment fold (EF) > 100). No significant enrichment in biological process was identified in this set of DAPs. The set of downregulated DAPs shared between ↑Osm and ↑Osm↓Temp (19 DAPs) was significantly enriched in several biological processes, including: FA biosynthetic (4/20; EF > 100) and metabolic processes (5/58; EF: 29.26), transsulfuration (2/6; EF: > 100), acetyl-CoA biosynthetic process (2/7; EF: 96.98) and tRNA aminoacylation for protein translation (3/35; EF: 29.09). Shared, downregulated molecular functions were enriched in ATP citrate synthase activity (2/2; EF: > 100), fatty-acyl-CoA synthase activity (2/2; EF > 100) and FA synthase activity (2/6: EF: > 100), aminoacyl-tRNA ligase activity (3/35; EF: 29.09) as well as small molecule (10/804; EF: 4.22) and anion binding (9/746; EF: 4.09). Specificities of unique proteomes awaken by ↑Osm and ↑Osm↓Temp are described in the following sections (**Quantitative proteome of ↑Osm-treated *Y. lipolytica* vs control** and **Quantitative proteome of ↓Temp↑Osm-treated *Y. lipolytica* vs control**). Selected DAPs with their abundance profiles averaged across all the samples, under all the treatment variants, are presented in a form of heat map (Fig. 6).

### Quantitative proteome of ↑Osm-treated Y. lipolytica vs control

**Fig. 7:**
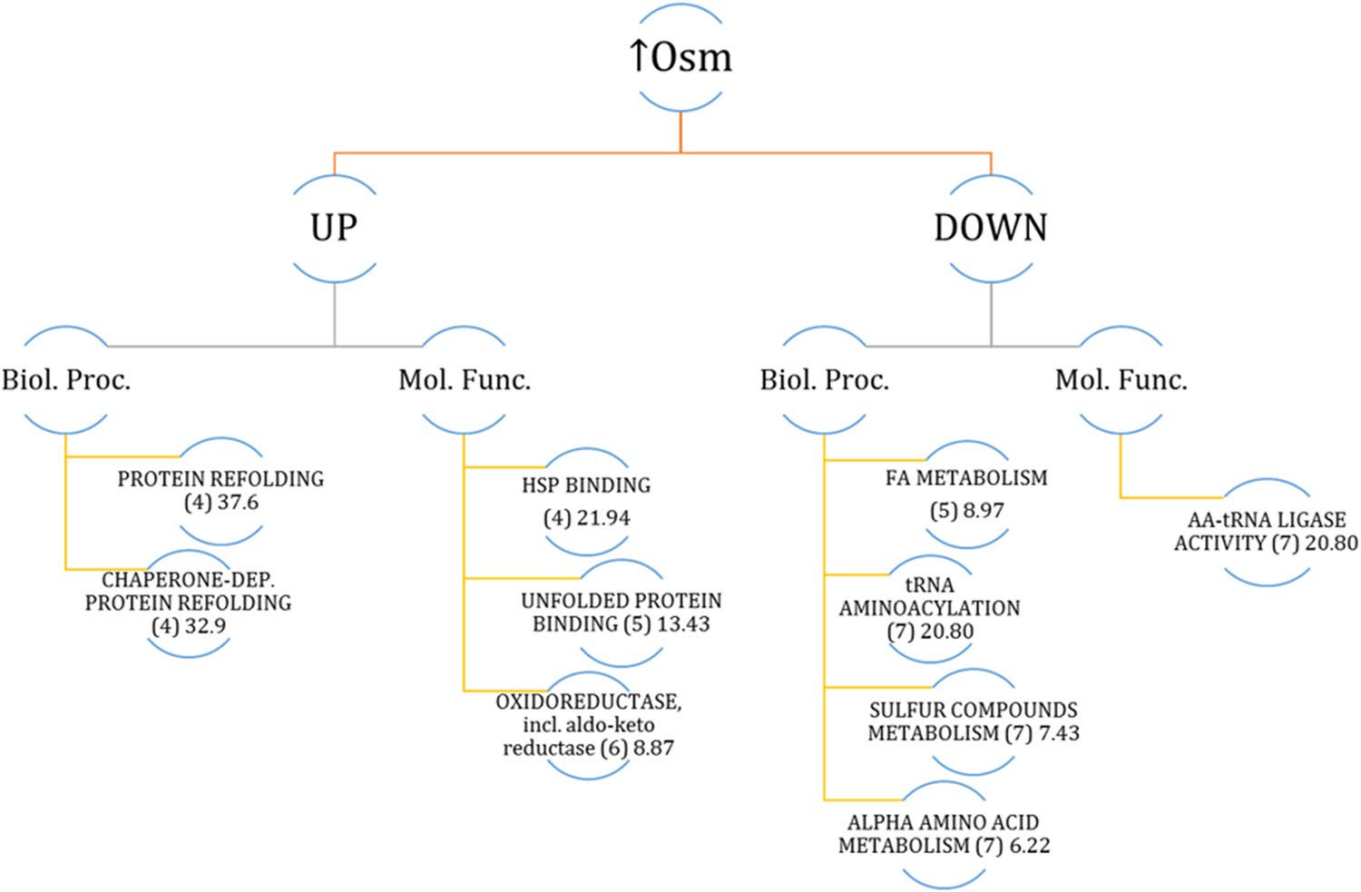
Significantly enriched biological processes (Biol. Proc.) and molecular functions (Mol. Func.) within a set of DAPs deregulated in response to ↑Osm. Only main, selected processes and functions are shown. Analysis was conducted with PANTHER statistical overrepresentation tool by feeding a list of DAPs’ YALI names. Numbers in brackets indicate the number of DAPs assigned to that group; the following numbers indicate enrichment fold

Implementation of high SORB-induced hyperosmolarity stress alone yielded 112 DAPs. The proteins were up-/down-regulated by a maximum fold factor of 11.08-/-3.16-FC (Supplemental Table S3). Functional annotation of DAPs was cross-referenced in PANTHER, GRYC and NCBI (via blastp similarity search), resulting in a carefully inspected landscape of biological processes and molecular functions deregulated under ↑Osm. PANTHER-based functional annotation and statistical overrepresentation analyses (Fig. 7) indicated that protein refolding was the dominant upregulated biological process. The refolding process was executed by the action of cytosolic (Ssa6/E35046, Ssa8/D22352, Hsp104/E27962, Hsp90/B15840), ER-localized (E25696, F00880) and mitochondrial (Hsp78/F12463; ISU1/B04928) (co-)chaperones induced by the factor of 1.23 to 3.8 (Fig. 6, Supplemental Table S3). In addition, oxidoreductases, incl. AKRs involved in polyols synthesis, were among the most upregulated DAPs (Gcy13/A15906, Gcy12/B07117, A19910, F24937, D08778), reaching average upregulation of > fourfold (compare Fig. 6). The other upregulated DAPs of high-relevance to the awaken stress response are proteins involved in oxidative stress response (glutathione peroxidase/E02310, glyoxalase/F00682), glycogen phosphorylase involved in mobilization of stored glycogen (F04169), highly upregulated proteins (> 2-FC) involved in mitochondrial respiration (D08602, F24409, D09933). Interestingly, DAPs involved in cellular membrane invagination and endocytosis (Pil1/C11341, D13442), vesicle transportation (B14102, F27379) and the major vacuolar protease Prb1 (A06435) were all upregulated, suggesting specific cellular reaction.

Molecular functions and biological processes downregulated under ↑Osm were primary represented by decreased abundance of amino acid-tRNA (aa-tRNA) synthetases specific to isoleucine (A00264), lysine (F16291), proline (E05027), leucine (E24607), asparagine (E05005), glutamate (E28468) and tryptophan (B08943) (Figs. 6 and 7). Likewise, proteins involved in ribosome biogenesis (E31625, F12661) and biosynthesis of AAs (Aro10/D06930, Bat2/F19910, Pro3/B14399), including sulfur compounds (CysK-Met25/D25168, MetB/C22088, B14509) were also identified within this group, altogether indicating significant attenuation of protein synthesis. In this regard, translation elongation factor gamma (eEF-1) was among the most downregulated DAPs under ↑Osm (D16467 FC − 1.63; and its homolog B12562 FC − 1.19). Furthermore, three karyopherins, involved in nuclear im-/export, were identified within this group of DAPs (E16324, F10098, D12144; Supplemental Table S3). The set of ↑Osm-driven downregulated DAPs was enriched in proteins representing several metabolic pathways involved in central carbon metabolism: FA metabolism (Fas1/B15059, Fas2/B19382, Fao1/B14014, Aal7/E20405, Pox3/D24750), glycolysis and TCA (Pyc1/C24101, Pyk1/F09185, Pgi/F07711, Acl1/E34793, Acl2/D24431). While not significantly enriched in the overrepresentation analysis (Fig. 7), several genes involved in cytoskeleton formation and remodeling were significantly downregulated in response to ↑Osm, including actin capping protein (C06325), tubulins (Tub1/E19360, Tub2/E00726) or a protein promoting actin folding (D13090). Within the downregulated DAPs, we also identified several proteins involved in proteolysis via vacuolar (C10494, F20592) or proteasomal (F28413, B02860, F09834) degradation.

### Quantitative proteome of ↓Temp↑Osm-treated Y. lipolytica vs control

**Fig. 8:**
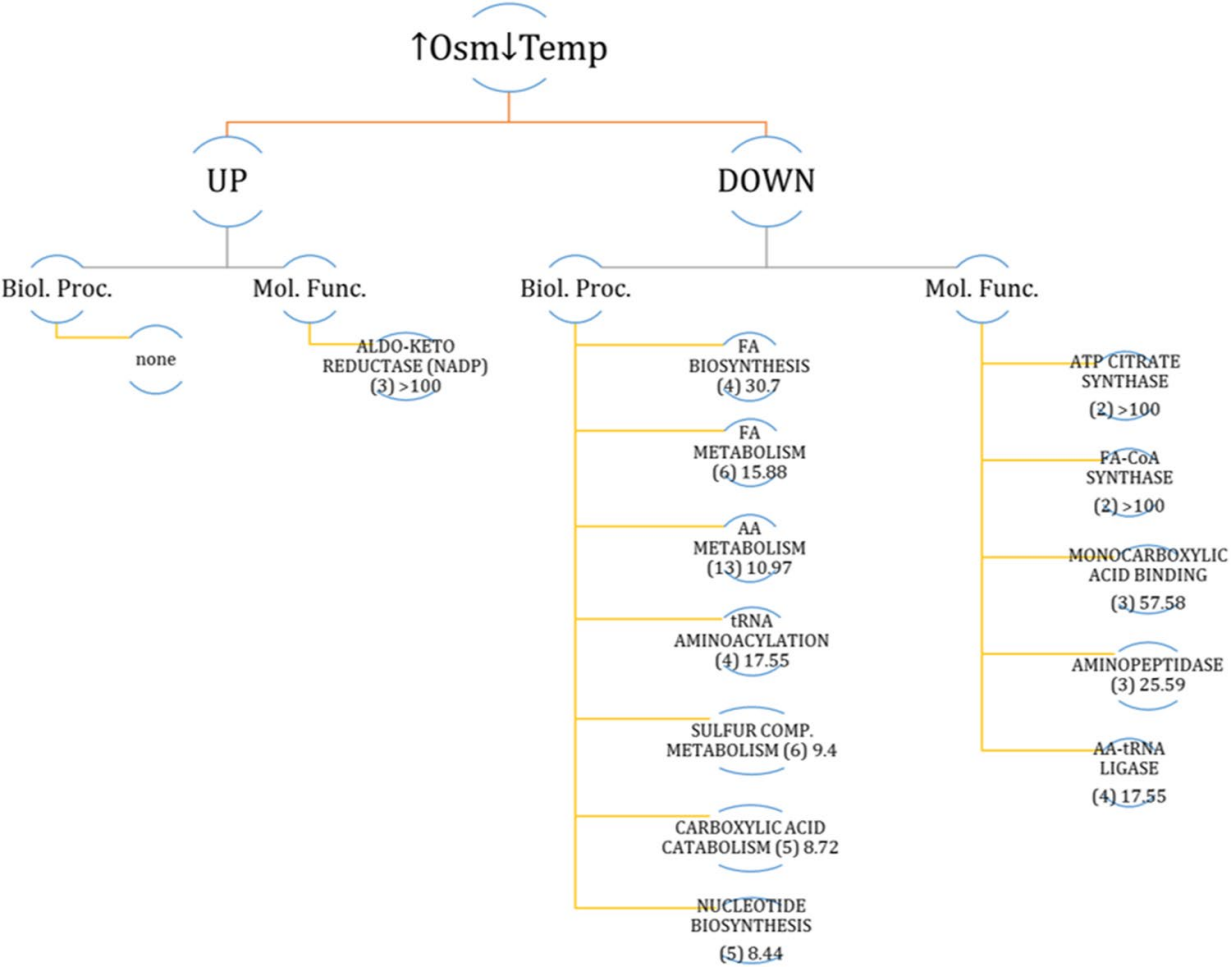
Significantly enriched biological processes (Biol. Proc.) and molecular functions (Mol. Func.) within a set of DAPs deregulated in response to ↑Osm↓Temp. Only main, selected processes and functions are shown. Analysis was conducted with PANTHER statistical overrepresentation tool by feeding a list of DAPs YALI names. Numbers in brackets indicate the number of DAPs assigned to that group; the following numbers indicate enrichment fold

Application of hyperosmolarity in combination with decreased temperature yielded 53 DAPs, which is less than half of what was identified for ↑Osm-treated culture. This observation suggests that additional execution of the low temperature treatment diminished impact of ↑Osm. The proteins were up-/down-regulated by a maximum fold factor of 13.3-/-1.91-FC (Supplemental Table S3). PANTHER-based functional annotation and statistical overrepresentation analyses (Fig. 8) rendered no significantly enriched biological processes within these upregulated DAPs. On the other hand, significantly enriched molecular function was represented by AKR activities, including Gcy12 (B07117) and Gcy13 (A15906), but also A19910 and D12386 (see also Fig. 6). Interestingly, within DAPs upregulated in response to ↓Temp↑Osm, we identified Atg8 (E02662), which is a multifunctional protein involved in autophagy-related processes, including selective nucleophagy, mitophagy, endoplasmic reticulum-specific autophagic process, autophagosomes formation and expansion, membrane invagination and filamentous growth regulation.

Molecular functions and biological processes downregulated under ↑Osm↓Temp were enriched in DAPs related to attenuation of protein synthesis. Primarily, we observed significantly decreased representation of aa-tRNA synthetases, specific to isoleucine (A00264), proline (E05027), arginine (E17985), asparagine (E05005) (downregulated by > 1.2-fold; Fig. 6, Supplemental Table S3). Furthermore, there was an additional panel of significantly downregulated aa-tRNA synthetases that did not meet the criterion of 1.2-FC (specific to aspartate (D22264), phenylalanine (E22979), methionine (F29843); not shown). Next, biosynthesis of several AAs was significantly enriched within these downregulated DAPs, represented by 4HPPDase/B21846 involved in tyrosine metabolism, Aat2/F29337 and Glt1/B19998 implicated in glutamate synthesis, glutamine synthetases /D13024 and A20108, and Arg4/D26367, CysK-Met25/D25168, MetB/C22088, playing roles in synthesis of arginine and sulfur-containing AAs (Fig. 6, Supplemental Table S3). As in the case of ↑Osm-induced response, the ↑Osm↓Temp proteome was characterized by significant downregulation of FA metabolism (Fas1/B15059, Fas2/B19382, Fao1/B14014, Aal7/E20405, Pox3/D24750), glycolysis and TCA (Pyc1/C24101, 6-phosphofructo-2-kinase/D21010, Acl1/E34793, Acl2/D24431). The majority of these DAPs were shared between the ↑Osm-downregulated set of proteins (Fig. 6, Supplemental Table S3).

### aa-tRNA synthetases promoter regions sequence search for common binding motif

**Fig. 9:**
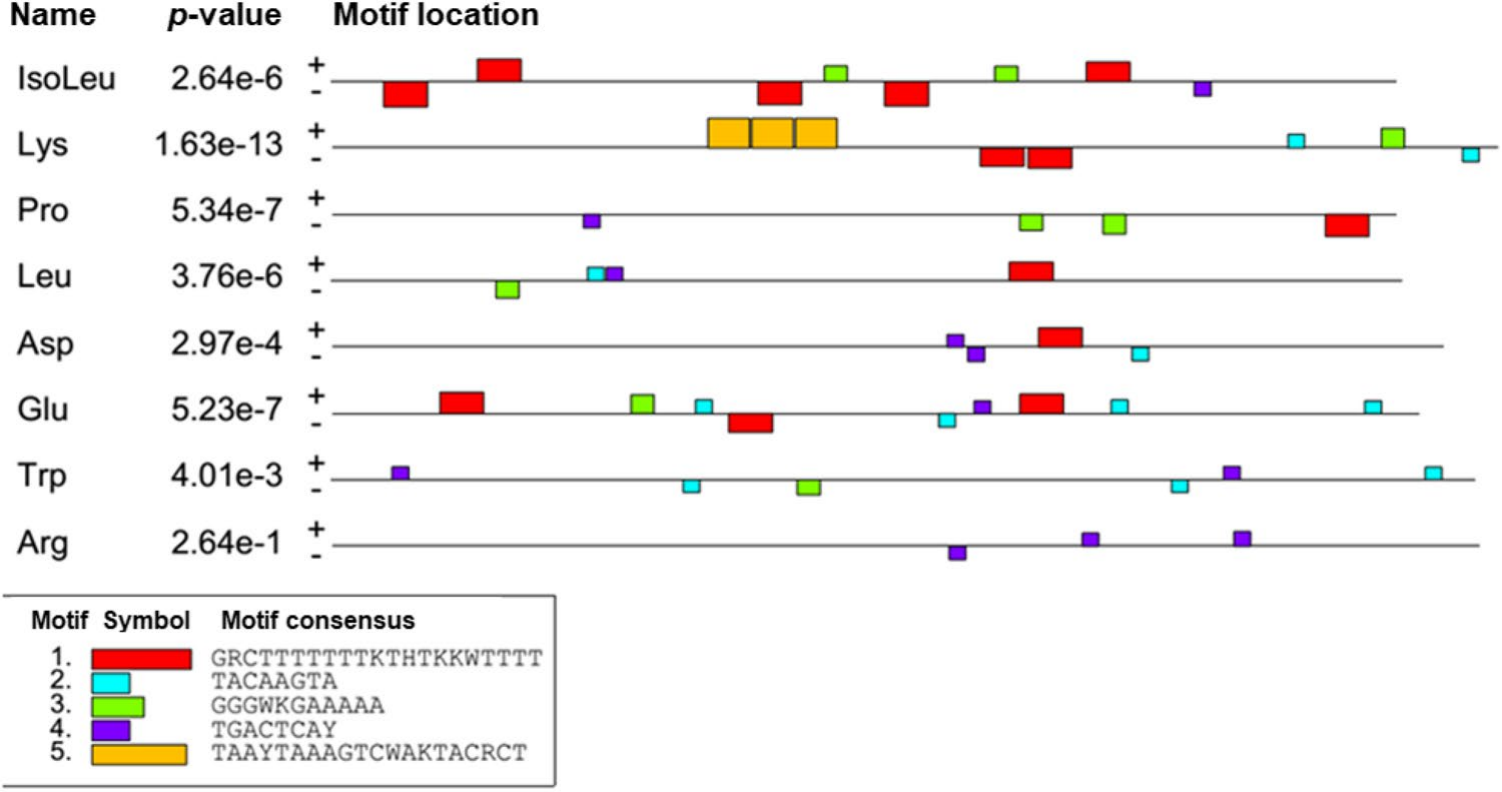
Visual representation of MEME motif search results conducted on 500 bp DNA sequence of genomic DNA upstream start codon of specific aa-tRNA synthetases identified in this work as downregulated DAPs. Name indicates specificity of a given ligase: isoleu/A00264, lys/F16291, pro/E05027, leu/E24607, asp/E05005, glu/E28468, tryp/B08943, arg/ E17985. Identified motifs are color coded according to the provided legend

Clearly marked downregulation of several aa-tRNA synthetases under ↑Osm and ↑Osm↓Temp treatments led us to hypothesis that maybe genes encoding these ligases are co-regulated. To test this, we extracted ~ 500 bp of genomic DNA regions upstream the ATG codon and analyzed them for occurrence of specific binding motifs. The results of this analysis are shown in Fig. 9. No single motif, uniformly present in all eight analyzed regions could be identified. The most frequent motif was #4 TGACTCAY (7/8), followed by #1 GRCTTTTTTTKTHTKKWTTTT (6/8) and #3 GGGWKGAAAAA (6/8).

## Discussion

Synthesis, maturation and secretion of rs-Prot is a complex biological process relying on multiple molecular functions. Depending on the biochemical characteristics of the rs-Prot and physiology of the host, different steps of the process may be limiting (Delic et al. 2013; Korpys-Woźniak et al. 2020; Swietalski et al. 2020). Nevertheless, high provision of HSPs (chaperones and foldases) and osmolytes within the producer cell seems to be a potentially generalizable strategy for enhancing rs-Prot production in bacteria and yeast (Oganesyan et al. 2007; Lazar et al. 2011). It is a widely recognized mechanism, that under exposure to different environmental stress factors, the cell increases the intracellular abundance of the protective agents to preserve delicate proteins and membranes from denaturation. This mechanism, among others, found survival and tolerance under adverse conditions. Development of a tolerant phenotype relies on active remodeling of cellular organization and thus requires significant transcriptional and translational effort. For example, osmotolerance in *Saccharomyces cerevisiae* is not reliant on sole intracellular accumulation of GLY, since osmotically conditioned cells washed free of GLY retain their capacity for growth on high salinity media. In contrast, blockage of protein synthesis inhibits the acquisition of the tolerant state, indicating a protein-mediated phenotype (Blomberg and Adler 1989; Blomberg 1995). Upregulation of HSP abundance is a part of general stress response, awaken under different stress conditions. Since HSPs execute their folding/protective role in response to stress conditions and as a part of a “regular” biosynthesis process, they constitute a link between the stress response and rs-Prots synthesis. The primary aim of this research was two-fold: (1) to settle if prolonged exposure to hyperosmolarity enhances synthesis of the rs-Prots in *Y. lipolytica*, (2) to get an insight into molecular background of the macroscopically observed physiological reaction of the cell to ↑Osm/↓Temp/↑Osm↓Temp treatments. Previously, we optimized thermal treatment conditions for enhanced synthesis of rs-Prots by *Y. lipolytica* (Kubiak et al. 2021). In the present study, we implemented that optimized treatment alone (↓Temp) or in combination with hyperosmolarity (↑Osm↓Temp). We were interested if application of the two treatment factors, presumably beneficial for rs-Prot synthesis, may exert combined/contradictory action, and what is the molecular background of it.

We induced the hyperosmotic conditions in *Y. lipolytica* batch cultures by abrupt addition of a non-utilizable by this yeast chemical compound—SORB. Such an approach allowed to stably maintain the stress conditions. In addition, since SORB is also inert for the rs-Prot, the risk of inactivating the reporter protein was eliminated. The ↑Osm stress was executed at the end of exponential growth phase. The treatment was followed by temporary growth arrest (3–5 h; Fig. 2), while the samples for molecular analyses were collected when the growth recommenced. Earlier studies showed that this growth-less adaptation stage is highly dynamic in terms of proteome remodeling, but the absolute rate of total protein synthesis is then roughly fourfold decreased (Blomberg 1995). In the present study, we were more interested in practical consequences of the newly developed organization of the cell that could potentially contribute to enhanced rs-Prot synthesis. Hence, it was reasonable to address this issue by analyzing the cells actively producing proteins after the recommencement of growth. For details on dynamic changes in the yeast osmo-proteome directly after osmotic shock (within 1 h), the reader is referred to excellent works by Blomberg (1995) and Soufi et al. (2009).

Considering the literature data, the observed growth arrest (3–5 h directly after the treatment execution) was expected in terms of occurrence, intensity and duration. The primary mediator of the osmostress response in yeast—Hog1—is known to regulate delay in the cell cycle to permit stress adaptation prior to its further progression (Clotet and Posas 2007). *S. cerevisiae* exposed to 1.4 Osm kg^−1^ by NaCl recovered growth within 1 h (Blomberg 1995). Here, we implemented over twofold higher osmolarity which had a significant limiting effect on the growth rates. In terms of metabolites synthesis, ↓Temp led to ~ 60% decrease in final titers of CA vs control, but the other typical compounds remained at unchanged levels. The same relates to GLY utilization profile shared between the ↓Temp and the control conditions. In this case, even though growth rate was significantly reduced, GLY was consumed at similar rates. Therefore, considering the observed physiological reaction of the cells, i.e. similar GLY consumption profile accompanied by limited growth and CA formation, but significantly elevated amounts of rs-Prot (Fig. 3), we infer that ↓Temp allows to channel carbon flux toward the rs-Prot synthesis. On the other hand, our proteomics data did not render any specific protein that was differentially abundant between the two conditions and could be potentially implicated in this redirection. This suggests that it was not regulated at proteomics level. Likewise, the gene expression analysis (Fig. 4) did not indicate any specific gene that could contribute to the enhanced quantities of the rs-Prot produced under ↓Temp. This indicates that these reactions must be regulated at some other than transcriptional level, or that some other genes are implicated, beyond the set of genes analyzed here.

Cultures subjected to ↑Osm (or ↑Osm↓Temp) were characterized by significantly elevated amounts of MAN (7x/ 8 × vs control) and ERY (2 × vs control), as expected. Proteomics and transcriptional data show that erythrose reductases Gcy12 and Gcy13, belonging to AKRs, were the key molecular identities, sharply responding to ↑Osm with enhanced expression. The omics data revealed several other AKR-type reductases (A19910, F24937, D08778) upregulated in response to ↑Osm, whose potential implication in the polyols synthesis could be verified in the following studies. Considering the very high disproportion in metabolic profiles developed under the control and the ↑Osm-treated variants, we looked for molecular bases of the metabolic fluxes redistribution. Apart from significant upregulation of the AKRs, we observed concerted downregulation of proteins involved in glycolysis, TCA and FA synthesis. Under hyperosmolarity, *S. cerevisiae* increases carbon fluxes toward GLY at the expense of carbon dioxide production (Blomberg 1995; Soufi et al. 2009). It was proposed that downregulation of glycolysis and ethanol/carbon dioxide synthesis is one of the prerequisites for increased formation of GLY, required for survival under the stress conditions in *S. cerevisiae* (Blomberg 1995; Soufi et al. 2009). The second prerequisite is transcriptional upregulation of *GPD1* (glycerol-3-phosphate dehydrogenase), the key enzyme required for GLY synthesis. It seems that a very similar mechanism operates in *Y. lipolytica*: (i) upregulation of the key enzymes involved in the polyols synthesis, which are known to be regulated at transcriptional level by ↑Osm (Trassaert et al. 2017), accompanied by (ii) downregulation in the other carbon fluxes, including glycolysis, TCA and FA biosynthesis. Proteins directly involved in this mechanism in *Y. lipolytica* were revealed and are indicated in Fig. 6 and Supplemental Table S3. Corresponding data regarding downregulation of glycolysis and TCA proteins (including Eno2, isocitrate dehydrogenase, Sdh1) and upregulation of AKRs were reported previously for salt-induced ↑Osm in *Y. lipolytica*, based on 2D-PAGE global proteomics (Yang et al. 2015).

In contrast to what was previously postulated (Fiedurek 1998; Oganesyan et al. 2007; Lazar et al. 2011; Kubiak et al. 2019), we did not observe any direct causative relationship between ↑Osm and ↑rs-Prot titer in *Y. lipolytica* (Fig. 3), at least when SORB was used as the osmolarity inducer at 3 Osm kg^−1^. The key modulator of osmo-stress response (Hog1) was not deregulated under ↑Osm/↑Osm↓Temp when compared to the control, at neither transcriptional (Fig. 4) nor protein level (Fig. 6, Supplemental Table S3). The same was reported previously with ↑Osm-treated *Y. lipolytica* studied by 2D-PAGE (Yang et al. 2015). It was expected, since the components of MAPK pathway are mainly responding to a specific inducer by their phosphorylation status rather than at transcriptional level (Soufi et al. 2009). Hence, considering a typical physiological reaction and some molecular evidence pointed hereafter, we speculate that Hog1 was indeed activated under the ↑Osm treatment, and its signaling cascade was initiated. An example of such a molecular evidence is high upregulation of *SKO1* (Fig. 4) encoding a transcription factor that is a direct downstream target of Hog1 (Gomar-Alba et al. 2013). Sko1 is an ATF/CREB repressor which is a substrate of Hog1 and is phosphorylated under osmotic stress. Such mechanism exemplifies one of nuclear/transcriptional functions of Hog1, executed via the Sko1 repressor. Transcriptional functions of Hog1 are also executed by several transcriptional activators, including Msn2/4, Smp1 and Hot1. Except for that, Hog1 also plays a cytoplasmic/non-transcriptional role in promoting stress tolerance by sequential activation of multiple targets. One of such targets is the Rck2 serine-threonine protein kinase (Teige et al. 2001). Since it does not respond to ↑Osm at transcriptional level, we have not seen it as DAP; however, our data suggest that we observed downstream physiological reaction of the Rck2’s activation. As proved by Teige et al. (2001), Hog1-driven activation of Rck2 is responsible for attenuation of protein synthesis in response to osmotic challenge through modification of translation elongation factor 2 (EF-2). It was shown that Rck2 regulates translation by phosphorylation of EF-2, leading to its decreased binding affinity to ribosomes and consequently to a lower level of protein synthesis (Teige et al. 2001). In the present study, the ↑Osm treatment decreased both titer and specific activity of the target rs-Prot (Fig. 3), although its gene expression was significantly increased (Fig. 4). These contradictory data from transcriptional and proteomics analysis are even further strengthened by significant upregulation of several HSPs and chaperones, which are known to assist protein synthesis, folding and maturation in *Y. lipolytica* (Celińska and Nicaud 2019). In addition, our recent study clearly indicated that co-overexpression of *Ssas* genes, identified as DAPs under ↑Osm (Figs. 4, 6, 7, Supplemental Table S3), leads to significant increase in titers of overproduced rs-Prots (Korpys-Woźniak et al. 2021). Here, even upon upregulation of the Ssas and the other chaperoning activities, the final yield of the rs-Prot under ↑Osm was lower. For most of eukaryotic organisms, the rapid inhibition of protein synthesis is an element of a stress response (Blomberg 1995; Simpson and Ashe 2012). As shown by Dunand-Sauthier et al. (2005) global protein synthesis is rapidly, but transiently, reduced in *S. cerevisiae* following exposure to osmotic stress, induced by KCl. Within 1 h, the cells restore normal global protein synthesis. Teige et al. (2001) showed that this inhibition is driven by Hog1-Rck2 cascade activation. Our data revealed several specific downstream mechanisms by which this downregulation of protein synthesis is executed. Primarily, we observed massive downregulation of aa-tRNA synthetases (Fig. 6, Supplemental Table S3). Their downregulation limits translation and renders the already transcribed tRNAs uncharged. The uncharged tRNAs are known potent regulators of both transcription (by direct interaction with promoter regions) and translation (by direct interaction with translation machinery) (Raina and Ibba 2014; Gomez and Ibba 2020). Except for downregulation of the aa-tRNA synthetases in both ↑Osm and ↑Osm↓Temp, we observed systemic downregulation of proteins involved in AAs biosynthesis, and downregulation of translation elongation factor gamma (its two homologs—D16467, B12562) (Fig. 6, Supplemental Table S3). This elongation factor could work as an interaction partner with uncharged tRNA. In addition, Hog1 is known to physically interact with Sfp1, the transcription factor that regulates ribosome biogenesis. Phosphorylated Sfp1 is recruited to the ribosome biogenesis genes (Sellam et al. 2019), but this mechanism is abolished under stress. Among DAPs, we identified several proteins significantly downregulated in response to ↑Osm and ↑Osm↓Temp which could be downstream consequences of Hog1-Sfp1 interaction. Based on all these observations, we infer that ↑Osm conditions decrease proteins biosynthesis in *Y. lipolytica* cells by several concerted mechanisms, including: (i) downregulation of aa-tRNA synthetases and (ii) translation elongation factor gamma, (iii) downregulation of AAs biosynthesis and (iv) ribosomes biogenesis. We speculate that the positive impact of the increased abundance of chaperones, HSPs and osmolytes does not outbalance the losses by the former phenomena. The observed proteomics profiles suggest that de novo synthesis of proteins is arrested, but the already synthesized polypeptides are subjected to chaperoning/folding activity. Similar patterns of elevated abundance of HSPs, including cytoplasmic Ssas, severe downregulation of translation elongation factor alpha and partial downregulation of genes involved in AAs and ribosomes biosynthesis, were observed in the formerly reported proteomics profile of osmostressed *Y. lipolytica* (Yang et al. 2015).

We found the concerted downregulation of so many aa-tRNA synthetases very intriguing. As a side question posed here, we asked if these genes can be co-regulated by sharing a common binding motifs in their regulatory regions. Furthermore, we were interested if these potential motifs are known to be specific to any of known transcription factors inflicted in stress response. Our analysis conducted on promoter regions (Fig. 9) revealed several motifs potentially driving the putative co-regulation. One of the most interesting was a motif TGACTCAY, present in 7 out of 8 analyzed regions. The motif shares high similarity with the ATF/CREB consensus sequence TGACGTCA (Rep et al. 2001), specific to i.a. Sko1, that was upregulated at transcriptional level in the present study (Fig. 4). While it is still highly speculative, the downregulated aa-tRNA synthetases could be the trans-targets of the TGACTCAY-specific transcription factors acting within osmostress response. Some DNA motifs potentially directing this regulation are shown in Fig. 9, to be further studied through detailed biochemical experimentation.

Another aspect of the awaken osmostress response observed in this study was localized to plasma membrane and cell wall. Among the upregulated DAPs responsive to ↑Osm, we found several proteins involved in eisosomes formation, membrane invagination and endocytosis, including Pil1 (C11341). Pil1 is the key component of eisosomes—specific structures distributed uniformly across the cell surface periphery, serving as endocytic hotspots. Considering the growth arrest that we observed in the cultures following ↑Osm/↑Osm↓Temp execution, the increased endocytosis was rather surprising. Therefore, we speculate that the increased endocytosis could be related to sequestration of membrane channels and transporters, which were targeted for degradation in the vacuole. What supports our statement is that: (i) Hog1 migrates to the cell periphery under ↑Osm, to close Fps1 aquaglyceroporin (Lee et al. 2013), (ii) AAs transporters are redundant at the cellular membrane under ↑Osm, as the yeast cells respond with depression of AA uptake after osmotic shock (Blomberg 1995), (iii) eisosomes are regulatable portals that govern both location and magnitude of membrane traffic into the cell; though it is not known if the elements building eisosomes are directly interacting with Hog1, (iv) among the upregulated DAPs we identified several vacuolar proteases, including the major vacuolar protease Prb1 (A06435). Results from 2D-PAGE followed by MS (Yang et al. 2015) showed that indeed upon ↑Osm one of the key potassium ion channels was severely downregulated, demonstrating its redundancy under the treatment. Likewise, upregulation of Prb1 (called there cerevisin) was observed in that study, which corresponds to our current observations. It would be very interesting to test this hypothesis by more detailed molecular biology research.

Finally, the proteomic profiles generated under ↑Osm suggest significant remodeling in the cytoskeleton and cell wall. Significant upregulation of UTR2 cell wall protein (B15510) indicates remodeling taking place at the cell wall, enhancing its rigidity. Soufi et al. (2009) showed that the cell wall integrity pathway is activated under osmostress by phosphorylation of Pkh1 and Ypk1/Ypk2 involved in maintenance of cell wall integrity during environmental stresses. In addition, we observed a number of DAPs down-regulated in response to ↑Osm that are involved in actin cables folding and actin-based transportation. As discussed earlier, increased external osmolarity causes a rapid loss of actin filament cables (Blomberg 1995). It was even suggested by that author that actin filament organization may act as an osmosensor responding to changes in turgor pressure and rapidly mediating this response to the general control points of the cell cycle. In relation to that, upregulation of Sko1, detected at transcriptional level in this study (Fig. 4), is known to limit filamentation (Su et al. 2013). Our microscopic observations of the cells following the ↑Osm treatment showed that the cells represented mainly ovoid/elongated ovoid morphotype (not shown).

In conclusion, we studied physiological, transcriptional and proteomic response of recombinant *Y. lipolytica* strains overproducing a heterologous rs-Prot subjected to ↑Osm, ↓Temp and ↑Osm↓Temp treatments. Our results indicate that hyperosmolarity is detrimental for the synthesis of rs-Prots, and molecular bases of this observation are described. Key molecular players involved redirection of carbon fluxes from CA and growth to polyols were revealed through the global proteomics analysis.

## Supplementary Information

Below is the link to the electronic supplementary material.

Supplementary file1 (PDF 2668 KB)

## Data Availability

All data accompanying this research are presented directly in the manuscript and supplementary materials. The mass spectrometry data were deposited to the ProteomeXchange Consortium via the MassIVE repository with the dataset identifier PXD029106.
