# Supplementary material for: Hyperosmolarity adversely impacts recombinant protein synthesis by Yarrowia lipolytica—molecular background revealed by quantitative proteomics
Source: Appl Microbiol Biotechnol. 2021 Dec 16;106(1):349–67. doi: 10.1007/s00253-021-11731-y (PMC8720085; doi:10.1007/s00253-021-11731-y)

Hyperosmolarity adversely impacts heterologous secretory protein synthesis by *Yarrowia lipolytica* – molecular background revealed by global quantitative proteomics

Applied Microbiology and Biotechnology

Monika Kubiak-Szymendera†, Bożena Skupień-Rabian‡, Urszula Jankowska‡, Ewelina Celińska*†

†Department of Biotechnology and Food Microbiology, Poznan University of Life Sciences, Wojska Polskiego 48, 60-627 Poznan, Poland

‡Proteomics and Mass Spectrometry Core Facility, Malopolska Centre of Biotechnology, Jagiellonian University, Gronostajowa 7a, 30-387 Krakow, Poland

*corresponding Author: ewelina.celinska@up.poznan.pl; ORCID: 0000-0001-8372-8459

Table S1 List of real-time PCR primers

Name	Primer sequence 5' → 3'
r-t_SoAMY_Fw	GTAACAACGTGGGAATCCGAAT
r-t_SoAMY_Rev	CCCTGGCCGTTCGAAGTAG
r-t_GCY12_Fw	TAACGGTAACGACCCCGTCTA
r-t_GCY12_Rev	AGCCGGACTCGTCAATGTCT
r-t_HSP12_Fw	AGGGCGAGTCTTACATTGATCAG
r-t_HSP12_Rev	TTTCCACCAAGGAAGTTGTTGA
r-t_HSP20_Fw	AAGCGGCGCCTTCGA
r-t_HSP20_Rev	GGCAGAGATGTTGTCTTCCTCAA
r-t_STI1_Fw	CCGCCATTGACCACTTCAC
r-t_STI1_Rev	AGGCGGATCTGTTGGAGTAGAG
r-t_GRP78_Fw	GCTTGACGCTAACGGAATTCTG
r-t_GRP78_Rev	TTGGAGAGACGGCCCTTGT
r-t_HSP104_Fw	CAGACTGTCTCGATCGGGTCTT
r-t_HSP104_Rev	GGAGAGACCAAGGAAAAGGAAAG
r-t_DNAJ_Fw	CCGTGAGACCTCCATCAACA
r-t_DNAJ_Rev	CGACACCCGCAGAATCG
r-t_Mhy1_Fw	CGGCGCCAGGAGCAT
r-t_Mhy1_Rev	GTCGCAGTTGAAAGGCTTCTCT
r-t_HSF1_Fw	GCCGCGGTTCTTCAAACATA
r-t_HSF1_Rev	TGCCACCCGTACATGTTGAG
r-t_TPS1_Fw	GCGTGCCCGTGTTCATG
r-t_TPS1_Rev	TGGAGTTGGAGAAGCCATTGT
r-t_TPS2_Fw	CCGGAACGCCATATGGATAA
r-t_TPS2_Rev	ATCGCCGAGCCACTTGTC
r-t_TPS3_Fw	TCCACCCTGACGAATTCTTCA
r-t_TPS3_Rev	TGATGGAGGCTGCACAATAGC
r-t_FMO1_Fw	ACCGAACCTGCCATGCA
r-t_FMO1_Rev	GCCGTCCTTTTTGTGCTCAA

r-t_SSA6_Fw	CCAATGACCAGGGAAACAGAA
r-t_SSA6_Rev	GCATCACCAATGAGTCGTTCA
r-t_SSA7_Fw	GAAGGACATCTCCACCAACCA
r-t_SSA7_Rev	TGGGCGGAAGAAGACAGAGT
r-t_HOG1_Fw	CCTGAAAATCTGCGACTTTGG
r-t_HOG1_Rev	TCGACACGTAGCCGGTCAT
r-t_TPI1_Fw	CAAGACCGAGGTGGTCATCTC
r-t_TPI1_Rev	AGACACGGTGGGCTTCTTCA
r-t_AHP1_Fw	CGAGATCGAGGGCAACGT
r-t_AHP1_Rev	AAGCTCCTTATCGGAGTGGAAA
r-t_SKN7_Fw	ATGGCCACGCCGACAA
r-t_SKN7_Rev	TCTGCACCTCGCCATTCTG
r-t_SKO1_Fw	TCCGTTCGAGCAGTCGTTT
r-t_SKO1_Rev	CGAGAGTCGAGGAGCTCCAT
r-t_RPL3_Fw	TGCACCGTTGTGCGAGTT
r-t_RPL3_Rev	GGTGAGCCTTCTTCTGCTTAAGAG
r-t_Kin2_Fw	TCCAAATTTCAGGACCAGTAAGG
r-t_Kin2_Rev	CGCGTGTCGTTGAAAGCTT

Table S2 Details of PCA analysis and hierarchical clustering of the proteome profiles

	Sample No	# Protein identified
CONTROL	1	2336
CONTROL	2	2242
CONTROL	3	2342
CONTROL	4	2425
TEMP	5	2260
TEMP	6	2325
TEMP	7	2246
TEMP	8	2247
OSM	9	2390
OSM	10	2505
OSM	11	2363
OSM	12	2333
TEMP_OSM	13	2348
TEMP_OSM	14	2118
TEMP_OSM	15	2437
TEMP_OSM	16	2378

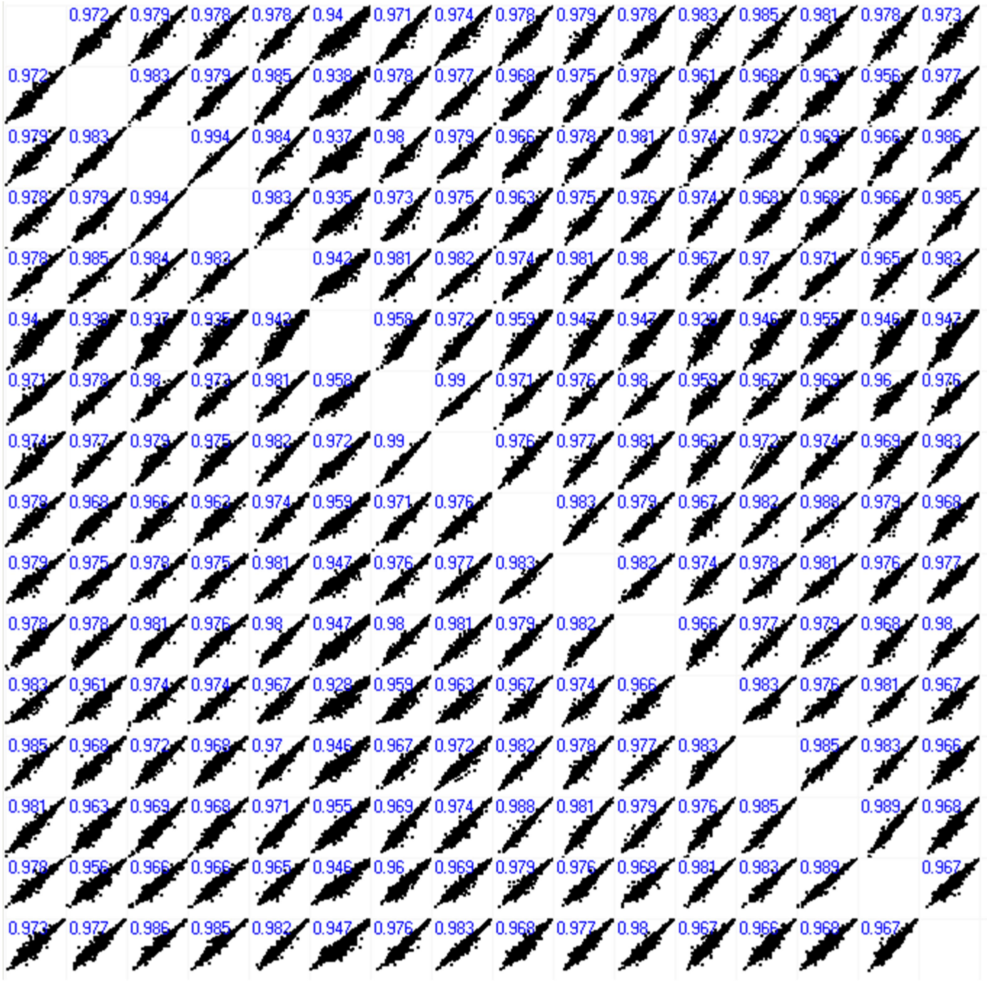

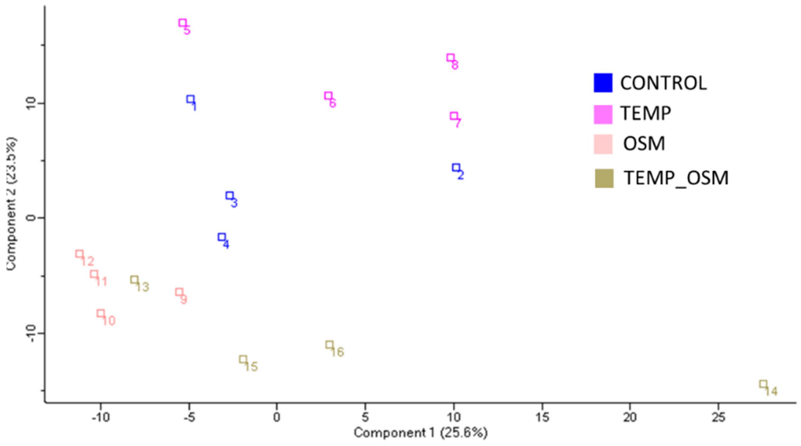

Table S3 Lists of all the DAPs with functional annotation and fold change (FC) of abundance

	↓Osm vs control					
**YALI NAME**	**other name**	**function**	**GRYC name/annotation**	**GRYC function**	**notes**	**FC**
YALI0C19096g			Hypothetical protein conserved in the *Yarrowia* clade			-3.16
YALI0_C06325g	HD domain-containing protein	Actin-capping protein, nucleolus		Actin capping protein – identified based on blastp. Sequence similarity	actin capping protein	-2.90
YALI0_B21846g	4-hydroxyphenylpyruvate dioxygenase	Oxidoreductase activity, involved in tyrosine metabolic process aromatic amino acid family catabolic process	Similar to uniprot|e9cwp5 *coccidioides immitis* tcrp 4-hydroxyphenylpyruvate dioxygenase 4hppd hpd hppdase	4-hydroxyphenylpyruvate dioxygenase		-2.85
YALI0_A12177g	TauD domain-containing protein	Oxygenase, ALPHA-KETOGLUTARATE-DEPENDENT SULFONATE DIOXYGENASE, involved in cellular catabolic process, sulfur compound metabolic process	Weakly similar to uniprot|Q12358 *Saccharomyces cerevisiae* YLL057C JLP1 Fe(II)-dependent sulfonate/alpha-ketoglutarate dioxygenase, involved in sulfonate catabolism for use as a sulfur source	Conserved hypothetical protein		-2.50
YALI0_A21439g	TauD domain-containing protein	Oxygenase, ALPHA-KETOGLUTARATE-DEPENDENT SULFONATE DIOXYGENASE, involved in cellular catabolic process, sulfur compound metabolic process	Similar to uniprot|Q7S5S1 *Neurospora crassa* NCU09800 putative dioxygenase	Dioxygenase. Putative		-2.28
YALI0_B04312g	Inositol-3-phosphate synthase	Isomerase, INOSITOL-3-PHOSPHATE SYNTHASE 1, involved in alcohol biosynthetic process, cellular carbohydrate biosynthetic process	INO1 Myo-inositol-1-phosphate synthase	Myo-inositol-1-phosphate synthase		-2.06
YALI0_F04620g			Similar to uniprot|Q6CCX8 *Yarrowia lipolytica* YALI0C05687g	Hypothetical protein of a 4-member gene family, conserved in the Yarrowia clade		-2.06
YALI0_F31735g	Ribonucleoside-diphosphate reductase	Oxidoreductase complex; involved in nucleotide biosynthetic process, carbohydrate derivative biosynthetic process	Highly similar to uniprot|q9p839 *candida albicans* rnr1 ribonucleotide reductase large subunit			-1.84
YALI0_D11125g		Replication protein	Similar to uniprot|q92374 *schizosaccharomyces pombe* replication factor-a protein 3 (single-stranded dna- binding protein p12 subunit)			-1.77
YALI0_B21428g	D-fructose-6-	Transaminase, GLUCOSAMINE--	Highly similar to uniprot|P14742 *Saccharomyces cerevisiae* YKL104C GFA1			-1.70

	phosphate amidotransferase	FRUCTOSE-6-PHOSPHATE AMINOTRANSFERASE, ISOMERIZING, involved in UDP-N-acetylglucosamine metabolic process, organophosphate metabolic process, protein N-linked glycosylation, phosphate-containing compound metabolic process	(ohnolog of YMR084W) Glutamine-fructose-6-phosphate amidotransferase			
YALI0_D16841g	MMS19 nucleotide excision repair protein	DNA REPAIR/TRANSCRIPTION PROTEIN MET18/MMS19, scaffold/adapter protein	Weakly similar to uniprot|P40469 *Saccharomyces cerevisiae* YIL128w MET18 involved in NER repair and RNA polymerase II transcription			-1.68
YALI0_E34793g	ATP citrate synthase	Transferase	Uniprot|q6c3h5 *yarrowia lipolytica* yali0e34793g acl1 atp citrate lyase		protein involved in glycolysis and TCA	-1.66
YALI0_D16467g	GST C-terminal domain-containing protein	ELONGATION FACTOR 1-GAMMA, translational elongation	Weakly similar to uniprot|P29547 *Saccharomyces cerevisiae* YPL048w CAM1 translation elongation factor eef1 gamma		translation elongation factor gamma (eEF-1)	-1.63
YALI0_B12562g		ELONGATION FACTOR 1-GAMMA, translational elongation			translation elongation factor gamma (eEF-1)	
YALI0_E31625g	U3 small nucleolar RNA-associated protein 22	NUCLEOLAR RNA-ASSOCIATED PROTEIN, RNA metabolism protein, involved in rrna processing ribonucleoprotein complex export from nucleus	Similar to uniprot|G8Y8Y3 *Millerozyma farinosa* Piso0_004494		protein involved in ribosome biogenesis	-1.57
YALI0_F14245g	RRM domain-containing protein	MKI67 FHA DOMAIN-INTERACTING NUCLEOLAR PHOSPHOPROTEIN, RNA binding, involved in maturation of LSU-rrna from tricistronic rrna transcript (SSU-rrna, 5.8S rrna, LSU-rrna)				-1.56
YALI0_E16324g	Importin N-terminal domain-containing protein	IMPORTIN N-TERMINAL DOMAIN-CONTAINING PROTEIN, invollve in protein import into nucleus	Similar to uniprot|P46970 *Saccharomyces cerevisiae* YJR132w NMD5 NAM7P interacting protein	Karyopherin. Putative	karyopherin. involved in nuclear im-/export	-1.56
YALI0_F21406g	Carbonic anhydrase	Dehydratase	Weakly similar to uniprot|p53615 *saccharomyces cerevisiae* nce103 nce3 ynl036w carbonic anhydrase			-1.55

YALI0_E19536g	SEC7 domain-containing protein	Golgi-specific brefeldin a-resistance guanine nucleotide exchange factor 1	Weakly similar to uniprot|p39993 saccharomyces cerevisiae yel022w (ohnolog of yjr031c) gea2 gdp/gtp exchange factor for arf			-1.51
YALI0_B15059g	FAS1, Fatty acid synthase subunit beta	Involved in long-chain fatty acid metabolic process, fatty acid biosynthetic process	Fas1 fatty acid synthase subunit beta, putative		Protein involved in fatty acid metabolism	-1.50
YALI0_D13090g	Phosducin domain-containing protein	Viral or transposable element protein, VIRAL IAP-ASSOCIATED FACTOR HOMOLOG	Similar to uniprot|Q12017 *Saccharomyces cerevisiae* YOR281c PLP2 IAP-associated factor Viaf1	Protein that interacts with the CCT complex to stimulate actin folding. Putative	A494ctin folding promotor protein	-1.49
YALI0_F10098g	Importin N-terminal domain-containing protein	Transporter activity, involved in regulation of protein transport, regulation of cellular localization, ribosomal subunit export from nucleus	Similar to uniprot|*p30822 saccharomyces cerevisiae* ygr218w crm1 nuclear export factor, exportin	Major karyopherin. Putative	Karyopherin. Involved in nuclear im-/export	-1.47
YALI0_B19162g	Aminotran_5 domain-containing protein	L-CYSTEINE DESULFHYDRASE, lyase	Similar to uniprot|O94021 *Candida albicans* Hypothetical 48.1 kda protein			-1.47
YALI0_B14014g	Alcohol oxidase	Oxidoreductase, GLUCOSE-METHANOL-CHOLINE GMC OXIDOREDUCTASE	FAO1 Fatty alkohol oxidase		Protein involved in fatty acid metabolism	-1.45
YALI0_B19382g	Fatty acid synthase subunit alpha		FAS2 Fatty acid synthase subunit alpha			-1.45
YALI0_E00726g	Tubulin beta chain	Involved in microtubule cytoskeleton organization, mitotic nuclear division	Highly similar to uniprot|p02557 *saccharomyces cerevisiae* yfl037w tub2 beta-tubulin		Tubulin	-1.45
YALI0_D24431g			ACL2 ATP citrate lyase		Protein involved in glycolysis and TCA	-1.44
YALI0_D24750g	POX3, Acyl-coenzyme A oxidase 3	Oxidoreductase in peroxisome, involved in lipid homeostasis, fatty acid beta-oxidation	Pox3 fatty-acyl coenzyme a oxidase		Protein involved in fatty acid metabolism	-1.41
YALI0_D06930g		Pyruvate decarboxylase	Similar to uniprot|q06408 *saccharomyces cerevisiae* ydr380w **aro10** phenylpyruvate decarboxylase, catalyzes decarboxylation of phenylpyruvate to phenylacetaldehyde		Protein involved in biosynthesis of amino acids	-1.41
YALI0_A00264g	Isoleucyl-tRNA	Ligase activity, involved in trna	Similar to uniprot|P09436 *Saccharomyces cerevisiae* YBL076c ILS1 isoleucyl-			-1.41

	synthetase	metabolic process, translational elongation, cellular amino acid metabolic process	trna synthetase			
YALI0_D12144g	Importin N-terminal domain-containing protein	Transporter activity, signal sequence binding, invovled in protein import into nucleus	Similar to uniprot|q06142 *saccharomyces cerevisiae* ylr347c kap95 karyopherin-beta singleton		Karyopherin. Involved in nuclear im-/export	-1.40
YALI0_F28413g	PCI domain-containing protein	Protease, ubiquitin proteasome pathway, 26S PROTEASOME NON-ATPASE REGULATORY SUBUNIT 3, involved in ubiquitin-dependent protein catabolic process	Similar to uniprot|P40016 *Saccharomyces cerevisiae* YER021w SUN2 26S proteasome regulatory subunit	Non-atpase regulatory subunit of the 26S proteasome lid	Protein involved in proteolysis via proteasomal degradation	-1.40
YALI0_E20405g		Ligase, LONG-CHAIN-FATTY-ACID--COA LIGASE	AAL7 Acyl/aryl-coa ligase		Protein involved in fatty acid metabolism	-1.35
YALI0_A06743g		Transferase, GLUTATHIONE S-TRANSFERASE-RELATED	Similar to uniprot|Q8NJZ8 *Emericella nidulans* Theta class glutathione S-transferase	Theta class glutathione S-transferase		-1.35
YALI0_B03630g		Reductase, RIBONUCLEOSIDE-DIPHOSPHATE REDUCTASE SMALL CHAIN	Highly similar to uniprot|Q9HEW8 *Emericella nidulans* Ribonucleotide reductase			-1.33
YALI0_A21417g	Beta_elim_lyase domain-containing protein	L-THREONINE ALDOLASE-RELATED, involved in cellular amino acid catabolic process, alpha-amino acid metabolic process, cellular amino acid biosynthetic process	Uniprot|Q6CG81 *Yarrowia lipolytica* YALI0A21417g CNT2 Hydroxytrimethyllysine aldolase, the second enzyme in the carnitine biosynthesis pathway	Hydroxytrimethyllysine aldolase		-1.33
YALI0_F20592g		ASPARTYL AMINOPEPTIDASE, metallopeptidase activity	Similar to uniprot|P14904 *Saccharomyces cerevisiae* YKL103c LAP4 aminopeptidase ysci precursor, vacuolar	Vacuolar aminopeptidase ysci	Protein involved in proteolysis via vacuolar degradation	-1.32
YALI0_F19910g	Branched-chain-amino-acid aminotransferase	Transaminase, BRANCHED-CHAIN-AMINO-ACID AMINOTRANSFERASE, involved in oxidation-reduction process, branched-chain amino acid biosynthetic process, alpha-amino acid metabolic process	Uniprot|Q6C120 *Yarrowia lipolytica* YALI0F19910g **BAT2** Branched-chain Amino acid Transaminase, cytoplasmic		Protein involved in biosynthesis of amino acids	-1.32
YALI0_E19360g	Tubulin alpha chain	Involved in microtubule	Highly similar to uniprot|p09733	Alpha-1 tubulin	**Tub1**.	-1.32

		cytoskeleton organization, mitotic nuclear division	*saccharomyces cerevisiae* yml085c (ohnolog of yml124c) tub1 alpha-1 tubulin		Tubulin	
YALI0_C10494g		Metallopeptidase activity, ASPARTYL AMINOPEPTIDASE	Similar to uniprot|P14904 *Saccharomyces cerevisiae* YKL103C LAP4 Vacuolar aminopeptidase	Vacuolar aminopeptidase ysci	Protein involved in proteolysis via vacuolar degradation	-1.31
YALI0_B07667g	N-(5'-phosphoribosyl)anthranilate isomerase	Isomerase, involved in alpha-amino acid metabolic process, aromatic amino acid family biosynthetic process, heterocycle biosynthetic process, cellular nitrogen compound biosynthetic process	Trp1 phosphoribosylanthranilate isomerase			-1.29
YALI0_F08591g		Acetyltransferase, GLUCOSE REPRESSIBLE PROTEIN MAK10, N-ALPHA-ACETYLTRANSFERASE 35, NATC AUXILIARY SUBUNIT	Weakly similar to uniprot|Q02197 *Saccharomyces cerevisiae* YEL053c MAK10 glucose-repressible protein			-1.28
YALI0_C24101g	Pyruvate carboxylase	Ligase activity, PYRUVATE CARBOXYLASE, MITOCHONDRIAL, involved in carbohydrate biosynthetic process, small molecule biosynthetic process, glucose metabolic process, monocarboxylic acid metabolic process	**PYC1** Pyruvate carboxylase		Karyopherin. Involved in nuclear im-/export	-1.27
YALI0_B02860g	26S proteasome regulatory subunit RPN1	Protease, Ubiquitin proteasome pathway, 26S PROTEASOME NON-ATPASE REGULATORY SUBUNIT, involved in proteasome-mediated ubiquitin-dependent protein catabolic process	Similar to uniprot|*P38764 Saccharomyces cerevisiae* YHR027C RPN1 Non-atpase base subunit of the 19S regulatory particle of the 26S proteasome	Non-atpase base subunit of the 19S RP of the 26S proteasome	Protein involved in proteolysis via proteasomal degradation	-1.26
YALI0_F09185g	PYK1 Pyruvate kinase	Involved in oxidation-reduction process, glycolytic process	**Pyk1** pyruvate kinase		Protein involved in glycolysis and tca	-1.26
YALI0_D25168g		Homocysteine/cysteine synthase	Similar to uniprot|p06106 *saccharomyces cerevisiae* ylr303w met25 o-acetylhomoserine sulfhydrylase	O-acetyl homoserine-o-acetyl serine sulfhydrylase	Cysk-met25. Playing role in synthesis of sulfur-	-1.26

					containing amino acids	
YALI0_F12661g	WD_REPEATS_REGION domain-containing protein	Pak1 interacting protein 1	Weakly similar to uniprot|p20484 *saccharomyces cerevisiae* ykl021c mak11 protein involved in an early, nucleolar step of 60s ribosomal subunit biogenesis		Protein involved in ribosome biogenesis	-1.26
YALI0_F16291g	Lysine--tRNA ligase	LYSYL-TRNA SYNTHETASE, aminoacyl-trna synthetase, involved in trna metabolic process, translational elongation, cellular amino acid metabolic process	Highly similar to uniprot|P15180 *Saccharomyces cerevisiae* YDR037w KRS1 lysyl-trna synthetase, cytosolic	Lysyl-trna synthetase. Cytosolic	Amino acid-trna (aa-trna) synthetases specific to Lysine	-1.25
YALI0_D10571g	Proliferating cell nuclear antigen	DNA polymerase processivity factor, DNA replication	Similar to uniprot|Q03392 *Schizosaccharomyces pombe* Proliferating cell nuclear antigen (PCNA)	Proliferating cell nuclear antigen		-1.24
YALI0_B14399g	Pyrroline-5-carboxylate reductase	PYRROLINE-5-CARBOXYLATE REDUCTASE, Proline biosynthesis	Similar to uniprot|Q12740 *Zalerion arboricola* **PRO3** Pyrroline-5-carboxylate reductase (P5C reductase)		Protein involved in biosynthesis of amino acids	-1.23
YALI0_E05027g	Prolyl-tRNA synthetase	Aminoacyl-trna synthetase, involved in trna metabolic process, translational elongation, cellular amino acid metabolic process	Similar to uniprot|P38708 *Saccharomyces cerevisiae* YHR020W Conserved protein of unknown function that may interact with ribosomes, putative prolyl-trna ligase		Amino acid-trna (aa-trna) synthetases specific to Proline	-1.23
YALI0_E24607g	Leucyl-tRNA synthetase	Aminoacyl-trna synthetase, involved in trna metabolic process, translational elongation, cellular amino acid metabolic process	Similar to uniprot|P26637 *Saccharomyces cerevisiae* YPL160w CDC60 leucine--trna ligase, cytosolic	Cytosolic leucyl trna synthetase	Amino acid-trna (aa-trna) synthetases specific to Leucine	-1.23
YALI0_E05005g	Asparaginyl-trna synthetase		Similar to uniprot|P38707 *Saccharomyces cerevisiae* YHR019c DED81 asparaginyl-trna-synthetase	Cytosolic asparaginyl-trna synthetase	Amino acid-trna (aa-trna) synthetases specific to Asparagine	-1.23
YALI0_B14509g	S-adenosylmethionine synthase	Nucleotidyltransferase	Highly similar to uniprot|q9p842 *candida albicans* casam2 s-adenosylmethionine synthetase 2	S-adenosylmethionine synthetase 2	Protein including sulfur	-1.22

					compounds	
YALI0_C22088g		CYSTATHIONINE GAMMA-SYNTHASE, Methionine biosynthesis, involved in alpha-amino acid metabolic process, cellular amino acid biosynthetic process, sulfur compound metabolic process	Similar to uniprot|P38716 *Saccharomyces cerevisiae* YHR112c similarity to cystathionine gamma-synthases		Metb, playing role in synthesis of sulfur-containing amino acids	-1.21
YALI0_E28468g	Glutamyl-tRNA synthetase	GLUTAMATE--TRNA LIGASE, CYTOPLASMIC, aminoacyl-trna synthetase	Similar to uniprot|P46655 *Saccharomyces cerevisiae* YGL245w Glutamyl-trna synthetase, cytoplasmic	Glutamyl-trna synthetase (glurs)	Amino acid-trna (aa-trna) synthetases specific to Glutamate	-1.21
YALI0_E13035g	Peptidase_M24 domain-containing protein	Protease, PROLIFERATION-ASSOCIATED PROTEIN 2G4	Similar to uniprot|Q09184 *Schizosaccharomyces pombe* SPAC23H4.09 curved DNA-binding protein (42 kda protein)	Curved DNA-binding protein		-1.21
YALI0_A12045g	6,7-dimethyl-8-ribityllumazine synthase	Transferase, involved in flavin biosynthesis/lumazine synthase	Similar to uniprot|p50861 *saccharomyces cerevisiae* yol143c rib4 6,7-dimethyl -8-ribityllumazine synthase	Lumazine synthase (dmrl synthase)		-1.21
YALI0_B19558g	TRM8, tRNA (guanine-N(7)-)-methyltransferase	RNA processing factor, involved in trna methylation				-1.21
YALI0_B02838g		Serine protease, DIPEPTIDYL PEPTIDASE 4-RELATED	Similar to uniprot|Q96VT7 *Aspergillus niger* Dipeptidyl aminopeptidase type IV	Dipeptidyl aminopeptidase type IV		-1.20
YALI0_E15642g	Replication protein A subunit					-1.20
YALI0_F09834g	26S proteasome regulatory subunit RPN2	Protease, proteasome-mediated ubiquitin-dependent protein catabolic process	Similar to uniprot|p32565 *saccharomyces cerevisiae* yil075c sen3 26s proteasome regulatory subunit	Subunit of the 26s proteasome	Protein involved in proteolysis via proteasomal degradation	-1.20
YALI0_B08943g	Tryptophanyl-tRNA synthetase	Aminoacyl-trna synthetase	Similar to uniprot|Q12109 *Saccharomyces cerevisiae* YOL097C WRS1 Cytoplasmic tryptophanyl-trna synthetase, aminoacylates tryptophanyl-trna	Cytoplasmic tryptophanyl-trna synthetase	Amino acid-trna (aa-trna) synthetases specific to Tryptophan	-1.20

YALI0_F07711g	Glucose-6-phosphate isomerase	Involved in pentose phosphate pathway/glycolysis	Highly similar to uniprot|p12709 *saccharomyces cerevisiae* ybr196c **pgi1** glucose-6-phosphate isomerase	Glucose-6-phosphate isomerase	Protein involved in glycolysis and tca	-1.20
YALI0_C19624g	ETR1, Enoyl-[acyl-carrier-protein] reductase, mitochondrial	Reductase, involved in fatty acid metabolic process	Etr1 2-enoyl thioester reductase, member of the medium chain dehydrogenase/reductase family			1.20
YALI0_A19888g		Meiotically up-regulated gene 191 protein	Similar to uniprot|a5dbk5 *meyerozyma guilliermondii* hypothetical protein pgug_00660			1.23
YALI0_E25696g		CO-CHAPERONE PROTEIN DJLA, chaperone activity	Similar to uniprot|Q9HGM9 *Schizosaccharomyces pombe* DNAJ domain protein	DNAJ domain protein	ER-localized (co)chaperone	1.23
YALI0_C03850g	RanBD1 domain-containing protein	Scaffold/adaptor protein, NUCLEAR PORE COMPLEX PROTEIN NUP50A-RELATED	Weakly similar to uniprot|P14907 *Saccharomyces cerevisiae* YJL041W NSP1 Essential component of the nuclear pore complex, which mediates nuclear import and export			1.28
YALI0_F00880g		Chaperone, DNAJ-LIKE-2, ISOFORM A	Similar to uniprot|P25491 *Saccharomyces cerevisiae* YNL064c YDJ1 mitochondrial and ER import protein	Type I HSP40 co-chaperone	ER-localized (co)chaperone	1.31
YALI0_F23551g		Transferase, THIOSULFATE SULFERTANSFERASE, involved in alpha-amino acid metabolic process, sulfur compound metabolic process	Similar to uniprot|Q875D2 *Podospora anserina*, similarity to thiosulfate sulfurtransferases	Thiosulfate sulfurtransferase		1.31
YALI0_C11341g		SPHINGOLIPID LONG CHAIN BASE-RESPONSIVE PROTEIN **PIL1**, involved in endocytosis protein phosphorylation, vesicle budding from membrane, cellular component assembly, negative regulation of protein kinase activity, membrane invagination	Highly similar to uniprot|Q12230 *Saccharomyces cerevisiae* YPL004C LSP1 Primary component of eisosomes, which are large immobile patch structures at the cell cortex associated with endocytosis	Primary component of eisosomes	Protein involved in cellular membrane invagination and endocytosis	1.31
YALI0_B09647g	Multifunctional fusion protein	Dehydrogenase, DELTA-1-PYRROLINE-5-CARBOXYLATE DEHYDROGENASE 1, ISOFORM A-RELATED, involved in heterocycle catabolic process, cellular amino acid catabolic process, alpha-amino acid metabolic process, organic cyclic compound	Similar to uniprot|P07275 *Saccharomyces cerevisiae* YHR037W PUT2 Delta-1-pyrroline-5-carboxylate dehydrogenase, mitochondrial precursor	Delta-1-pyrroline-5-carboxylate dehydrogenase		1.32

		catabolic process				
YALI0_B14102g		Non-motor actin binding protein, DREBRIN-LIKE PROTEIN, involved in actin filament polymerization, regulation of actin filament polymerization	Weakly similar to uniprot|P15891 *Saccharomyces cerevisiae* YCR088w ABP1 Actin binding protein		Protein involved in cellular membrane invagination and endocytosis	1.36
YALI0_E02310g	Glutathione peroxidase	Peroxidase, GLUTATHIONE PEROXIDASE-LIKE PEROXIREDOXIN 1-RELATED	Highly similar to uniprot|P40581 *Saccharomyces cerevisiae* YIR037W (ohnolog of YKL026C) HYR1 glutathione peroxidase	Thiol peroxidase	Protein involved in oxidative stress response	1.36
YALI0_D01980g		Benzil reductase ((s)-benzoin forming) irc24	Similar to uniprot|p40579 *saccharomyces cerevisiae* yir035c similarity to human corticosteroid 11-beta- dehydrogenase	Cytoplasmic short-chain dehydrogenase/reductase		1.38
YALI0_B09471g	PHB domain-containing protein	Transporter, STOMATIN-LIKE PROTEIN 2, MITOCHONDRIAL	Similar to uniprot|O60121 *Schizosaccharomyces pombe* Stomatin family protein			1.38
YALI0_F16379g		Membrane traffic protein, DYNAMIN-RELATED PROTEIN DNM1	Similar to uniprot|P54861 *Saccharomyces cerevisiae* YLL001w DNM1 dynamin-related protein	Dynamin-related gtpase involved in mitochondrial organization		1.41
YALI0_D22352g		HEAT SHOCK PROTEIN 70KDA, involved in chaperone-mediated protein folding, cellular response to unfolded protein, 'de novo' protein folding	**SSA8**/SSA-B HSP70 protein		Cytosolic (co)chaperone	1.44
YALI0_D08778g	Epimerase domain-containing protein	Dehydratase, NAD DEPENDENT EPIMERASE/DEHYDRATASE	Similar to uniprot|P53183 *Saccharomyces cerevisiae* YGL039w	Oxidoreductase shown to reduce carbonyl compounds to chiral alcohols	Aldo-keto reductase involved in polyol synthesis	1.44
YALI0_F22187g		Dauer up-regulated-related	Some similarities with uniprot|q7s1u2 *neurospora crassa* ncu09057.1 hypothetical protein			1.44
YALI0_F00682g	DJ-1_PfpI domain-containing protein	Glutathione-independent glyoxalase hsp31-related	Similar to uniprot|q04432 *saccharomyces cerevisiae* ydr533c hypothetical protein	Methylglyoxalase that converts methylglyoxal to d-lactate	Protein involved in oxidative stress response	1.46
YALI0_B00924g			Hypothetical protein conserved in the *Yarrowia* clade			1.46

YALI0_C07502g			SLA2 Sla2p cytoskeleton assembly control protein	Sla2p cytoskeleton assembly control protein		1.48
YALI0_D06501g	2,5-diamino-6-(5-phospho-D-ribosylamino)pyrimidin-4(3H)-one reductase	Reductase, DIHYDROFOLATE REDUCTASE FAMILY PROTEIN	Similar to uniprot|P33312 *Saccharomyces cerevisiae* YBR153w RIB7 5-amino-6-(5-phosphoribosylamino)uracil reductase	Diaminohydroxyphoshoribosylaminopyrimidine deaminase		1.50
YALI0_C04213g		BYSTIN, involved in rrna processing	Similar to uniprot|P38333 *Saccharomyces cerevisiae* YBR247c ENP1 effects N-glycosylation	Protein associated with U3 and U14 snornas		1.50
YALI0_F27379g	Sec2p domain-containing protein	Guanyl-nucleotide exchange factor, RAB GUANINE NUCLEOTIDE EXCHANGE FACTOR SEC2, involved in vesicle fusion to plasma membrane	Weakly similar to uniprot|O13930 *Schizosaccharomyces pombe* hypothetical serine-rich protein	Weakly similar to uniprot|O13930 *Schizosaccharomyces pombe* hypothetical serine-rich protein	Vesicle transportation	1.53
YALI0_A06435g		Serine protease, PEPTIDASE S8	Similar to uniprot|P09232 *Saccharomyces cerevisiae* YEL060C (ohnolog of YOR003W) **PRB1** protease B, vacuolar	Vacuolar proteinase B with H3 N-terminal endopeptidase activity	Major vacuolar protease **PRB1**	1.54
YALI0_E27962g	Sigma-54 factor interaction domain-containing protein	HEAT SHOCK PROTEIN 104, protease, involved in cellular response to heat	Highly similar to uniprot|P31539 *Saccharomyces cerevisiae* YLL026w HSP104 heat shock protein	Hsp that cooperates with Ydj1p (Hsp40) and Ssa1p (Hsp70)	Cytosolic (co)chaperone	1.59
YALI0_B15840g	Aha1_N domain-containing protein	Heat shock protein 90 hsp90 co-chaperone aha-1	Similar to uniprot|q12449 *saccharomyces cerevisiae* ydr214w aha1 co-chaperone that binds to hsp82p and activates its atpase activity	Co-chaperone that binds hsp82p and activates its atpase activity	Cytosolic (co)chaperone	1.60
YALI0_F04169g	Alpha-1,4 glucan phosphorylase	Glycogen phosphorylase	Similar to uniprot|p06738 *saccharomyces cerevisiae* ypr160w gph1 glycogen phosphorylase	Glycogen phosphorylase required for the mobilization of glycogen		1.62
YALI0_E02024g			Hypothetical protein conserved in the *Yarrowia* clade			1.67
YALI0_E33297g		Transcription cofactor, PIRIN-RELATED	Similar to uniprot|Q873I9 *Neurospora crassa* B9B11.100 related to pirin			1.68
YALI0_D13442g	ENTH domain-containing protein	Involved in endocytosis, vesicle budding from membrane, membrane invagination	Similar to uniprot|p47160 *saccharomyces cerevisiae* yjr125c ent3 cytoskeletal adaptor	Protein containing an n-terminal epsin-like domain	Protein involved in cellular membrane invagination	1.75

					and endocytosis	
YALI0_F12463g		Protease, HEAT SHOCK PROTEIN 78, MITOCHONDRIAL, involved in cellular response to heat	Similar to uniprot|P33416 *Saccharomyces cerevisiae* YDR258C **HSP78** Oligomeric mitochondrial matrix chaperone		Mitochondrial (co)chaperone	1.77
YALI0_E35046g		HEAT SHOCK PROTEIN 70KDA, involved in chaperone-mediated protein folding, cellular response to unfolded protein, 'de novo' protein folding	**SSA6**/SSA-D HSP70 protein		Cytosolic (co)chaperone	1.80
YALI0_F06688g		Uncharacterized protein yjbq-related	Highly similar to uniprot|o14155 *schizosaccharomyces pombe* spac4a8.02c. Gene			1.81
YALI0_D16775g			Similar to uniprot|P38884 *Saccharomyces cerevisiae* YHR198c			1.85
YALI0_B10362g		Primary active transporter, MITOCHONDRIAL IMPORT RECEPTOR SUBUNIT TOM70	Similar to uniprot|Q9HEG7 *Neurospora crassa* tom70 Mitochondrial precursor protein import receptor			1.86
YALI0_B15510g	Glycosidase	Glycosyl hydrolase-related	Similar to uniprot|p32623 *saccharomyces cerevisiae* yel040w utr2 cell wall protein			1.90
YALI0_F24937g	Epimerase domain-containing protein	Dehydratase, NAD DEPENDENT EPIMERASE/DEHYDRATASE	Similar to uniprot|P53111 *Saccharomyces cerevisiae* YGL157w ARI1 NADPH-dependent aldehyde reductase	NADPH-dependent aldehyde reductase	Aldo-keto reductase involved in polyol synthesis	1.95
YALI0_B08272g	PH domain-containing protein	Cytoskeletal signaling protein slm1	Some similarities with uniprot|p08965 *schizosaccharomyces pombe* meiosis protein mei2			1.98
YALI0_F11099g			Hypothetical protein conserved in the *Yarrowia* clade			2.02
YALI0_A19910g		Aldo/keto reductase	Similar to uniprot|p47137 *saccharomyces cerevisiae* yjr096w putative xylose and arabinose reductase		Aldo-keto reductase involved in polyol synthesis	2.05
YALI0_B21692g			Similar to uniprot|A2RA32 *Aspergillus niger* short- chain dehydrogenase/reductase			2.07
YALI0_D09427g		Short-chain dehydrogenase/reductase pkfc	Weakly similar to uniprot|o74959 *schizosaccharomyces pombe* hypothetical short chain dehydrogenase			2.17
YALI0_D08602g		PROTEIN FMP25, MITOCHONDRIAL, involved in mitochondrial respiratory chain complex III assembly	Some similarities with uniprot|Q08023 *Saccharomyces cerevisiae* YLR077w		Protein involved in mitochondrial respiration	2.20

YALI0_F28083g	**NOG2**	Nucleolar gtp-binding protein 2	Similar to uniprot|p53742 *saccharomyces cerevisiae* ynr053c nog2 gtpase			2.24
YALI0_F24409g	HIG1 domain-containing protein	RESPIRATORY SUPERCOMPLEX FACTOR 2, MITOCHONDRIAL, involved in mitochondrial cytochrome c oxidase assembly	Weakly similar to uniprot|P53721 *Saccharomyces cerevisiae* YNR018w		Protein involved in mitochondrial respiration	2.27
YALI0_D09933g	Alternative oxidase	Oxidase, involved in aerobic respiration	Similar to uniprot|q8j0i8 *yarrowia lipolytica* yali0e00814g aox1	Alternative oxidase	Protein involved in mitochondrial respiration	2.32
YALI0_D18403g	ATG26, Sterol 3-beta-glucosyltransferase	Sterol 3-beta-glucosyltransferase	Ugt51/atg26 udp-glucose:sterol glucosyltransferase			3.03
YALI0_B07117g	Aldo_ket_red domain-containing protein	Aldo/keto reductase	Gcy12 galactose-induced protein of aldo/keto reductase family		Aldo-keto reductase involved in polyol synthesis	3.53
YALI0_B04928g	ISU1, Iron sulfur cluster assembly protein 1, mitochondrial	Chaperone, IRON-SULFUR CLUSTER ASSEMBLY ENZYME NIFU HOMOLOG, involved in cellular iron ion homeostasis	Similar to uniprot|Q03020 *Saccharomyces cerevisiae* YPL135W (ohnolog of YOR226C) ISU1 strong similarity to nitrogen fixation protein	Conserved protein of the mitochondrial matrix	Mitochondrial (co)chaperone	3.80
YALI0_A15906g	Aldo_ket_red domain-containing protein	Aldo/keto reductase	Gcy13 galactose-induced protein of aldo/keto reductase family	Gtpase	Aldo-keto reductase involved in polyol synthesis	11.08

↑Osm↓Temp vs control							
**YALI NAME**	**other name**	**function**	**GRYC name/annotation**	**GRYC function**	**notes**	**FC ↑Osm**	**FC ↑O+↓T**
YALI0_B21846g	4-hydroxyphenylpyruvate dioxygenase	Oxygenase, involved in tyrosine metabolic process, aromatic amino acid family catabolic process	Similar to uniprot|e9cwp5 *coccidioides immitis* tcrp 4-hydroxyphenylpyruvate dioxygenase 4hppd hpd hppdase	4-hydroxyphenylpyruvate dioxygenase	Biosynthesis of amino acids. 4hppdase involved in tyrosine metabolism	-2.85	-1.91
YALI0_B19998g	Glutamine amidotransferase type-2 domain-containing protein	Fad nadph dehydrogenase/oxidoreductase	Similar to uniprot|q12680 *saccharomyces cerevisiae* ydl171c **glt1** glutamate synthase (napdph) (gogat)		Biosynthesis of amino acids, implicated in glutamate synthesis	-1.76	-1.75
YALI0_B12166g	**MBF1**, Putative multi-protein-binding factor 1	Endothelial differentiation-related factor 1	**Mbf1** putative multi-protein binding factor 1			-1.51	-1.70
YALI0_E34793g	ATP citrate synthase	Transferase	**Acl1** atp citrate lyase		Protein involved in glycolysis and tca	-1.66	-1.62
YALI0_B04312g	Inositol-3-phosphate synthase	Inositol-3-phosphate synthase 1	**Ino1** myo-inositol-1-phosphate synthase			-2.06	-1.54
YALI0_B08030g	**LIP3**	Carboxylic ester hydrolase	**Lip3** carboxylesterase/lipase type b			-1.22	-1.48
YALI0_B15059g	**FAS1,** Fatty acid synthase subunit beta	Fatty acid synthase subunit beta	**Fas1** fatty acid synthase subunit beta, putative		Protein involved in fatty acid metabolism	-1.50	-1.47
YALI0_B14014g	Alcohol oxidase	Glucose-methanol-choline gmc oxidoreductase	**Fao1** fatty alkohol oxidase		Protein involved in fatty acid metabolism	-1.45	-1.45
YALI0_D13024g	Gln-synt_C domain-containing protein	Ligase, TYPE-1 GLUTAMINE SYNTHETASE 2	Similar to uniprot|Q96WJ7 *Blumeria graminis* Glutamine synthetase (Glutamate--ammonia ligase)		Glutamine synthetase	-1.21	-1.41
YALI0_E19899g	AB hydrolase-1 domain-containing protein	2-succinyl-6-hydroxy-2,4-cyclohexadiene-1-carboxylate synthase	Weakly similar to uniprot|q7rzl5 *neurospora crassa* ncu00350.1			-1.31	-1.39
YALI0_B17402g	Adenylosuccinate lyase	Involved in purine ribonucleotide biosynthetic process, AMP metabolic process	Highly similar to uniprot|Q05911 *Saccharomyces cerevisiae* YLR359w ADE13 adenylosuccinate lyase			-1.23	-1.39

YALI0_D24750g	**POX3**, Acyl-coenzyme A oxidase 3	Oxidoreductase in peroxisome, involved in lipid homeostasis, fatty acid beta-oxidation	**Pox3** fatty-acyl coenzyme a oxidase	Protein involved in fatty acid metabolism	-1.41	-1.39
YALI0_E20405g		Long-chain-fatty-acid--coa ligase	**Aal7** acyl/aryl-coa ligase		-1,35	-1.38
YALI0_D16841g	MMS19 nucleotide excision repair protein	Scaffold/adaptor protein, DNA REPAIR/TRANSCRIPTION PROTEIN MET18/MMS19, involved in iron-sulfur cluster assembly, protein maturation	Weakly similar to uniprot|P40469 *Saccharomyces cerevisiae* YIL128w MET18 involved in NER repair and RNA polymerase II transcription		-1.68	-1.38
YALI0_D24431g			**ACL2** ATP citrate lyase	Protein involved in glycolysis and TCA	-1.44	-1.36
YALI0_A00264g	Isoleucyl-tRNA synthetase	Ligase activity, involved in trna metabolic process, translational elongation, cellular amino acid metabolic process	Similar to uniprot|P09436 *Saccharomyces cerevisiae* YBL076c ILS1 isoleucyl-trna synthetase	Amino acid-trna (aa-trna) synthetases specific to Isoleucine	-1.41	-1.35
YALI0_B19382g	Fatty acid synthase subunit alpha		**FAS2** Fatty acid synthase subunit alpha		-1.45	-1.35
YALI0_A20108g	Glutamine-dependent NAD(+) synthetase	Ligase, GLUTAMINE-DEPENDENT NAD(+) SYNTHETASE	Highly similar to uniprot|P38795 *Saccharomyces cerevisiae* YHR074w QNS1 Putative glutamine-dependent NAD(+) synthetase	Glutamine synthetase	-1.28	-1.34
YALI0_D21890g	Alpha-mannosidase	Alpha-mannosidase 2c1	Similar to uniprot|p22855 *saccharomyces cerevisiae* ygl156w ams1 alpha-mannosidase singleton<br>Vacuolar alpha mannosidase		-1.14	-1.32
YALI0_F10857g	**POX2** Acyl-coenzyme A oxidase 2	Oxidoreductase, ACYL-COENZYME A OXIDASE-LIKE PROTEIN	**POX2** Fatty-acyl coenzyme A oxidase		-1.32	-1.32
YALI0_A08077g	Serine/threonine-protein phosphatase	Protein phosphatase, SERINE/THREONINE PROTEIN PHOSPHATASE	Highly similar to uniprot|P20654 *Emericella nidulans* serine-threonine protein phosphatase PP1		-1.26	-1.32
YALI0_F20592g		Metalloprotease, ASPARTYL AMINOPEPTIDASE	Similar to uniprot|P14904 *Saccharomyces cerevisiae* YKL103c LAP4 aminopeptidase ysci precursor, vacuolar	Protein involved in proteolysis via vacuolar degradation	-1.32	-1.31
YALI0_A21417g	Beta_elim_lyase domain-containing protein	Aldolase, L-THREONINE ALDOLASE-RELATED	**CNT2** Hydroxytrimethyllysine aldolase, the second enzyme in the carnitine biosynthesis pathway		-1.33	-1.31

YALI0_C24101g	Pyruvate carboxylase	Ligase, PYRUVATE CARBOXYLASE, MITOCHONDRIAL	**PYC1** Pyruvate carboxylase	Protein involved in glycolysis and TCA	-1.27	-1.30
YALI0_C10494g		Metalloprotease, ASPARTYL AMINOPEPTIDASE	Similar to uniprot|P14904 *Saccharomyces cerevisiae* YKL103C LAP4 Vacuolar aminopeptidase		-1.31	-1.27
YALI0_C01859g	Methylmalonate-semialdehyde dehydrogenase (CoA acylating)	Dehydrogenase	Similar to uniprot|q02253 *rattus norvegicus* methylmalonate-semialdehyde dehydrogenase (acylating), mitochondrial precursor (mmsdh)		-1.12	-1.27
YALI0_D26367g	Arginosuccinase	Argininosuccinate lyase	Highly similar to uniprot|p04076 *saccharomyces cerevisiae* yhr018c **arg4** arginosuccinate lyase	Playing role in synthesis of arginine	-1.15	-1.27
YALI0_E07271g		Ligase, CARBOXYLASE:PYRUVATE/ACETYL-COA/PROPIONYL-COA CARBOXYLASE	**URC3** Urea carboxylase and allophanate hydrolase activities involved in uracile catabolism		-1.40	-1.27
YALI0_D21010g	6PF2K domain-containing protein	Carbohydrate phosphatase, 6-PHOSPHOFRUCTO-2-KINASE/FRUCTOSE-2,6-BISPHOSPHATASE	Weakly similar to uniprot|Q06137 *Saccharomyces cerevisiae* YLR345w similarity to PFK26P and other 6- phosphofructo-2-kinases	6-phosphofructo-2-kinase	-1.14	-1.26
YALI0_D25168g		Homocysteine/cysteine synthase	Similar to uniprot|p06106 *saccharomyces cerevisiae* ylr303w met25 o-acetylhomoserine sulfhydrylase	Cysk-met25, playing role in synthesis of sulfur-containing amino acids	-1.26	-1.26
YALI0_E05027g	Prolyl-tRNA synthetase	Bifunctional glutamate/proline--trna ligase	Similar to uniprot|p38708 *saccharomyces cerevisiae* yhr020w conserved protein of unknown function that may interact with ribosomes, putative prolyl-trna ligase	Amino acid-trna (aa-trna) synthetases specific to proline	-1.23	-1.25
YALI0_E17985g	Arginyl-tRNA synthetase	Arginine--trna ligase, mitochondrial-related	Similar to uniprot|q05506 *saccharomyces cerevisiae* ydr341c (ohnolog of yhr091c) arginyl-trna synthetase, cytosolic	Amino acid-trna (aa-trna) synthetases specific to arginine	-1.16	-1.24
YALI0_C24420g		Elongation factor 1-gamma	Similar to uniprot|p29547 *saccharomyces cerevisiae* ypl048w cam1 translation elongation factor eef1 gamma homologue		-1.15	-1.24
YALI0_C22088g		Lyase, CYSTATHIONINE GAMMA-SYNTHASE	Similar to uniprot|P38716 *Saccharomyces cerevisiae* YHR112c similarity to cystathionine gamma-synthases	Metb, playing role in synthesis of sulfur-containing amino acids	-1.21	-1.23
YALI0_E19184g		Protease, XAA-PRO AMINOPEPTIDASE 1	Similar to uniprot|Q8RY11 *Arabidopsis thaliana* AT3g05350/T12H1_32, putative X-pro dipeptidase		-1.18	-1.23
YALI0_F29337g	Aspartate aminotransferase	Aspartate transaminase	Similar to uniprot|p23542 *saccharomyces cerevisiae* ylr027c **aat2** aspartate aminotransferase	Biosynthesis of amino acids, implicated in glutamate synthesis	-1.17	-1.23
YALI0_F24717g	RRM domain-	RNA splicing factor, RRM	Weakly similar to uniprot|C5JP18 *Ajellomyces dermatitidis*		-1.19	-1.23

	containing protein	DOMAIN-CONTAINING PROTEIN	(Blastomyces dermatitidis) BDBG_04101 Grp1p, RNP domain-containing protein			
YALI0_E05005g	Asparaginyl-tRNA synthetase	Aminoacyl-trna synthetase, ASPARAGINE--TRNA LIGASE, CYTOPLASMIC	Similar to uniprot|P38707 *Saccharomyces cerevisiae* YHR019c DED81 asparaginyl-trna-synthetase	Amino acid-trna (aa-trna) synthetases specific to Asparagine	-1.23	-1.21
YALI0_C10901g	Thymidylate synthase	Methyltransferase, THYMIDYLATE SYNTHASE	Highly similar to uniprot|P06785 *Saccharomyces cerevisiae* YOR074c CDC21 thymidylate synthase		-1.17	-1.21
YALI0_F08283g	Nudix hydrolase domain-containing protein	Hydrolase, PEROXISOMAL NADH PYROPHOSPHATASE NUDT12	Weakly similar to uniprot|P53164 *Saccharomyces cerevisiae* YGL067w NPY1 NADH pyrophosphatase I		-1.16	-1.21
YALI0_D08976g	PCI domain-containing protein	26S PROTEASOME NON-ATPASE REGULATORY SUBUNIT 11, involved in ubiquitin-dependent protein catabolic process, proteasome assembly	Similar to uniprot|Q12377 *Saccharomyces cerevisiae* YDL097c RPN6 subunit of the regulatory particle of the proteasome		-1.16	-1.21
YALI0_B00726g	**DYS1** Deoxyhypusine synthase	Deoxyhypusine synthase	Highly similar to uniprot|p38791 *saccharomyces cerevisiae* yhr068w dys1 deoxyhypusine synthase	**Tub2**, tubulin	-1.18	-1.20
YALI0_E02662g	**ATG8** Autophagy-related protein 8	Non-motor microtubule binding protein, GAMMA-AMINOBUTYRIC ACID RECEPTOR-ASSOCIATED PROTEIN-LIKE 2	Highly similar to uniprot|P38182 *Saccharomyces cerevisiae* YBL078c AUT7 essential for autophagy	Multifunctional protein involved in autophagy-related processes, including selective nucleophagy, mitophagy, endoplasmic reticulum-specific autophagic process, autophagosomes formation and expansion, membrane invagination and filamentous growth regulation	1.10	1.35
YALI0_F23551g		Sulfurtransferase	Similar to uniprot|q875d2 *podospora anserina*, similarity to thiosulfate sulfurtransferases<br>Thiosulfate sulfurtransferase		1.31	1.36
YALI0_D12386g	Epimerase domain-	Nad dependent epimerase/dehydratase	Similar to uniprot|p53111 *saccharomyces cerevisiae* ygl157w ari1 nadph-dependent aldehyde reductase	Aldo-keto reductase	1.39	1.43

	containing protein						
YALI0_E02024g			Hypothetical protein conserved in the *Yarrowia* clade			1.67	1.67
YALI0_A19910g		Aldo/keto reductase	Similar to uniprot|p47137 *saccharomyces cerevisiae* yjr096w putative xylose and arabinose reductase		Aldo-keto reductase involved in polyol synthesis	2.05	1.74
YALI0_B11132g		Nuclear transport factor 2	Weakly similar to uniprot|p34232 *saccharomyces cerevisiae* ykl186c mtr2 mrna transport protein			1.34	1.95
YALI0_D00451g			Hypothetical protein conserved in the *Yarrowia* clade			2.34	2.17
YALI0_C21065g			Some similarities with uniprot|P47011 *Saccharomyces cerevisiae* YJL137C (ohnolog of YKR058W) GLG2 self-glucosylating initiator of glycogen synthesis			1.80	2.46
YALI0_B07117g	Aldo_ket_red domain-containing protein	Aldo-keto reductase family 1 member a1	**Gcy12** galactose-induced protein of aldo/keto reductase family			3.53	3.71
YALI0_A15906g	Aldo_ket_red domain-containing protein	Aldo/keto reductase	**Gcy13** galactose-induced protein of aldo/keto reductase family	Gtpase		11.08	9.27
YALI0_E34485g	Metallophos domain-containing protein	METALLOPHOS DOMAIN-CONTAINING PROTEIN; phosphodiesterase	Hypothetical protein conserved in the *Yarrowia* clade			2.14	13.30

Table S4 Details of Venn diagram analysis

UP [Osm] and [Osm + Temp]:	UP [Osm + Temp]:	DWN [Osm] and [Osm + Temp]:	DWN [Osm + Temp]:	DWN [Osm]:	UP [Osm]:
YALI0_F23551g	YALI0_E02662g	YALI0_B21846g	YALI0_B19998g	YALI0C19096g	YALI0_C19624g
YALI0_E02024g	YALI0_D12386g	YALI0_B04312g	YALI0_B12166g	YALI0_C06325g	YALI0_A19888g
YALI0_A19910g	YALI0_B11132g	YALI0_D16841g	YALI0_B08030g	YALI0_A12177g	YALI0_E25696g
YALI0_B07117g	YALI0_D00451g	YALI0_E34793g	YALI0_D13024g	YALI0_A21439g	YALI0_C03850g
YALI0_A15906g	YALI0_C21065g	YALI0_B15059g	YALI0_E19899g	YALI0_F04620g	YALI0_F00880g
	YALI0_E34485g	YALI0_B14014g	YALI0_B17402g	YALI0_F31735g	YALI0_C11341g
		YALI0_B19382g	YALI0_A20108g	YALI0_D11125g	YALI0_B09647g
		YALI0_D24431g	YALI0_D21890g	YALI0_B21428g	YALI0_B14102g
		YALI0_D24750g	YALI0_F10857g	YALI0_D16467g	YALI0_E02310g
		YALI0_A00264g	YALI0_A08077g	YALI0_E31625g	YALI0_D01980g
		YALI0_E20405g	YALI0_C01859g	YALI0_F14245g	YALI0_B09471g
		YALI0_A21417g	YALI0_D26367g	YALI0_E16324g	YALI0_F16379g
		YALI0_F20592g	YALI0_E07271g	YALI0_F21406g	YALI0_D22352g
		YALI0_C10494g	YALI0_D21010g	YALI0_E19536g	YALI0_D08778g
		YALI0_C24101g	YALI0_E17985g	YALI0_D13090g	YALI0_F22187g
		YALI0_D25168g	YALI0_C24420g	YALI0_F10098g	YALI0_F00682g
		YALI0_E05027g	YALI0_E19184g	YALI0_B19162g	YALI0_B00924g
		YALI0_E05005g	YALI0_F29337g	YALI0_E00726g	YALI0_C07502g
		YALI0_C22088g	YALI0_F24717g	YALI0_D06930g	YALI0_D06501g
			YALI0_C10901g	YALI0_D12144g	YALI0_C04213g
			YALI0_F08283g	YALI0_F28413g	YALI0_F27379g
			YALI0_D08976g	YALI0_A06743g	YALI0_A06435g
			YALI0_B00726g	YALI0_B03630g	YALI0_E27962g
				YALI0_F19910g	YALI0_B15840g
				YALI0_E19360g	YALI0_F04169g
				YALI0_B07667g	YALI0_E33297g
				YALI0_F08591g	YALI0_D13442g
				YALI0_B02860g	YALI0_F12463g
				YALI0_F09185g	YALI0_E35046g
				YALI0_F12661g	YALI0_F06688g
				YALI0_F16291g	YALI0_D16775g
				YALI0_D10571g	YALI0_B10362g
				YALI0_B14399g	YALI0_B15510g
				YALI0_E24607g	YALI0_F24937g
				YALI0_B14509g	YALI0_B08272g
				YALI0_E28468g	YALI0_F11099g
				YALI0_E13035g	YALI0_B21692g
				YALI0_A12045g	YALI0_D09427g
				YALI0_B19558g	YALI0_D08602g
				YALI0_B02838g	YALI0_F28083g
				YALI0_E15642g	YALI0_F24409g
				YALI0_F09834g	YALI0_D09933g
				YALI0_B08943g	YALI0_D18403g
				YALI0_F07711g	YALI0_B04928g

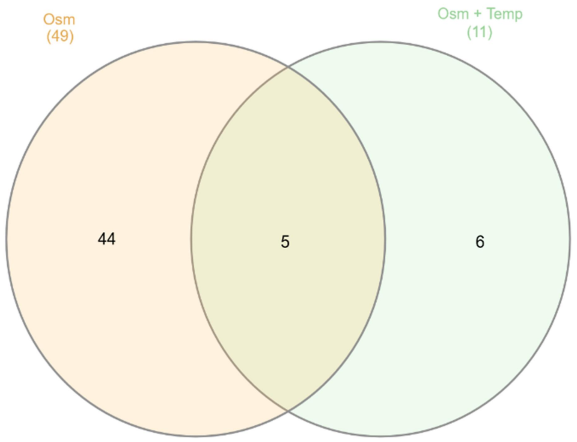

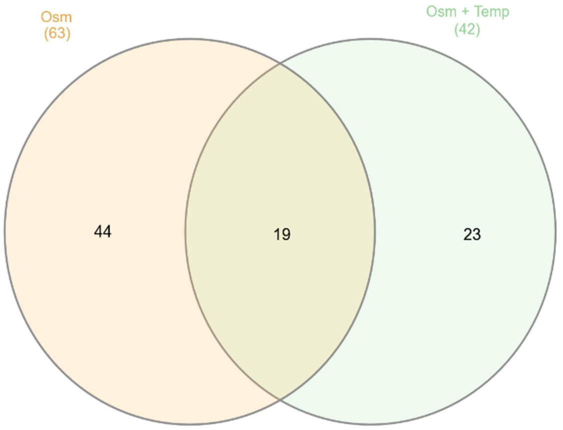

Supplement: Supplementary file 1 — Supplementary file1 (PDF 2668 KB) [file 253_2021_11731_MOESM1_ESM.pdf]
